# ALKBH3 suppresses ischemia/reperfusion‐induced PANoptosis by regulating the ZBED6/STAT1/AIM2 axis through m^1^A demethylation

**DOI:** 10.1002/ctm2.70632

**Published:** 2026-03-12

**Authors:** Hongtao Diao, Chunlei Wang, Yuting Xiong, Qiaoyue Zhao, Xinyue Zhang, Xiaohui Qi, Yuan Zou, Jiaxuan Li, Linghua Zeng, Wei Si, Feng Zhang, Ping Pang, Ning Wang, Yu Bian, Baofeng Yang

**Affiliations:** ^1^ Department of Pharmacology College of Basic Medical Sciences Jilin University Changchun China; ^2^ Department of Pharmacology the State Key Laboratory of Frigid Zone Cardiovascular Diseases (SKLFZCD) Key Laboratory of Cardiovascular Research Ministry of Education, College of Pharmacy, Harbin Medical University Harbin China; ^3^ Shanghai Frontiers Science Research Center for Druggability of Cardiovascular Noncoding RNA Institute for Frontier Medical Technology College of Chemistry and Chemical Engineering Shanghai University of Engineering Science Shanghai China

**Keywords:** AIM2, ALKBH3, m^1^A modification, PANoptosis

## Abstract

**Background:**

Myocardial ischemia/reperfusion (I/R) injury induces an intense inflammatory response and involves multiple cell death pathways. PANoptosis, an integrated cell death process involving pyroptosis, apoptosis and necroptosis, is a major driver of cardiomyocyte loss during I/R injury. However, the epitranscriptomic control of PANoptosis is poorly understood.

**Methods:**

We investigated the role of ALKBH3, an mRNA N^1^‐methyladenosine (m^1^A) demethylase, in the regulation of cardiomyocyte PANoptosis using hypoxia/reoxygenation models in vitro and murine I/R models in vivo. Integrated transcriptomic and m^1^A epitranscriptomic profiling identified downstream targets. Loss‐ and gain‐of‐function studies of ALKBH3, AIM2, ZBED6 and STAT1 (siRNA or plasmid overexpression) were coupled with assessments of cell death phenotypes, inflammasome activity and gene expression. Molecular interactions and transcriptional/translational regulation were examined using co‐immunoprecipitation, chromatin immunoprecipitation (ChIP) and dual‐luciferase reporter assays.

**Results:**

Cardiomyocyte‐restricted ALKBH3 overexpression mitigates I/R injury in vivo. Mechanistically, ALKBH3 acts as a key suppressor of PANoptosis by inhibiting AIM2. ALKBH3 demethylates m^1^A onZBED6 mRNA, enhancing ZBED6 translation and limiting cardiomyocyte PANoptosis. Although ZBED6 does not bind directly to the AIM2 promoter, it physically interacts with STAT1, a transcriptional activator of AIM2, and represses STAT1‐driven AIM2 expression. ZBED6 overexpression reduces AIM2 levels and PANoptosis, whereas AIM2 knockout attenuates the exacerbation of cardiac injury and PANoptosis induced by ALKBH3 silencing.

**Conclusions:**

These findings identify the ALKBH3/ZBED6/STAT1/AIM2 signalling axis that epitranscriptomically breaks cardiomyocyte PANoptosis, highlighting a tractable therapeutic target that limits cell death and improves myocardial outcomes after I/R.

## Graphical abstract

1. ALKBH3 alleviates myocardial ischemia/reperfusion injury by suppressing PANoptosis.
2. ALKBH3 promotes ZBED6 translation via mRNA m^1^A demethylation.
3. ZBED6 promotes the interaction between STAT1 and NEDD4L to facilitate STAT1 ubiquitination and subsequent degradation.
4. The degradation of STAT1 restricts AIM2 transcription, effectively blocking the downstream PANoptosis signalling cascade.

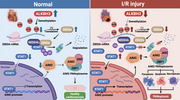

## 1 INTRODUCTION

Myocardial ischemia/reperfusion (I/R) injury is a paradoxical phenomenon in which the restoration of blood flow to the ischemic myocardium causes additional cellular damage beyond that caused by the initial ischemic injury.^1^, ^2^ Although timely reperfusion therapy, such as percutaneous coronary intervention and thrombolysis, is essential for salvaging the myocardium and limiting infarct size in patients with myocardial infarction, reperfusion triggers a complex cascade of pathological responses, including excessive inflammation,^3^, ^4^ oxidative stress,^5^ intracellular calcium overload,^6^, ^7^ mitochondrial dysfunction^8^, ^9^ and multiple forms of programmed cell death,^10^, ^11^ which together drive irreversible cardiomyocyte death, adverse ventricular remodeling and progressive cardiac dysfunction.^12^ Despite extensive research on the pathogenesis of I/R injury, effective therapeutic interventions that directly prevent or mitigate these downstream consequences are lacking. Elucidating the molecular mechanisms and regulatory networks underlying myocardial I/R injury is therefore crucial for identifying novel therapeutic targets and developing cardioprotective strategies.

Epigenetic RNA modifications function as critical molecular switches in cardiovascular diseases, particularly in myocardial I/R injury. Among these, N^1^‐methyladenosine (m^1^A), a dynamic and reversible RNA modification, has emerged as a key regulator of RNA metabolism, cellular stress responses and disease progression.^13^, ^14^, ^15^ m^1^A modification occurs in multiple RNA classes, including tRNA, mRNA, rRNA and lncRNA, and exerts context‐dependent regulatory effects under physiological and pathological conditions.^16^, ^17^ Unlike many other RNA markers, m^1^A introduces a positive charge at the Watson–Crick interface, altering the RNA secondary structure, modulating stability and translational efficiency and reshaping protein‐RNA interactions.^18^, ^19^, ^20^ Dysregulated m^1^A expression has been linked to tumorigenesis,^21^ neurodegeneration^22^ and immune dysregulation.^23^, ^24^ Although it is increasingly recognized in diverse diseases, the role of m^1^A in myocardial injury, particularly I/R, remains insufficiently defined. Functionally, m^1^A has been implicated in the control of oxidative stress, inflammatory signalling and DNA repair^25^, ^26^ processes that are central to I/R pathogenesis. ALKBH3 (a member of the AlkB family of Fe(II)/α‐KG‐dependent dioxygenases), a well‐characterized mRNA m^1^A eraser,^27^, ^28^, ^29^ remodels RNA‐modification landscapes under pathological stress. However, whether ALKBH3‐mediated m^1^A demethylation determines cardiomyocyte fate during ischemic stress remains unclear, underscoring the need to delineate its mechanistic contribution to cardiomyocyte death and myocardial injury.

Multiple programmed cell death pathways—including apoptosis,^30^, ^31^ necroptosis,^32^, ^33^ pyroptosis^34^, ^35^, ^36^ and ferroptosis^37^, ^38^, ^39^ have been implicated in cardiomyocyte loss and subsequent cardiac dysfunction.^40^ The crosstalk between these pathways shapes both the magnitude and outcome of myocardial injury. Recent studies have described PANoptosis,^41^, ^42^, ^43^ an inflammatory programmed cell‐death program that integrates pyroptotic, apoptotic and necroptotic components, as an important driver of I/R injury, particularly during robust immune activation and inflammasome assembly.^44^, ^45^, ^46^ Our preliminary findings suggest that ALKBH3‐mediated m^1^A dynamics critically regulate PANoptotic signalling during I/R, with AIM2 (absent in melanoma 2) emerging as a key downstream effector. AIM2 is a cytosolic DNA sensor and inflammasome initiator that bridges innate immune activation with pyroptotic signalling.^47^, ^48^, ^49^ Its dysregulation has been associated with exacerbated cardiac inflammation and cell death under stress.^50^, ^51^ However, the upstream epitranscriptomic control of AIM2 and PANoptosis during I/R remains unclear. The role of the m^1^A demethylase ALKBH3 in modulating PANoptosis has not been fully elucidated, representing a critical gap in our understanding of the regulation of cell death in ischemic heart diseases.

In this study, we studied the contribution of m^1^A to myocardial I/R injury by focusing on the ALKBH3/AIM2 signalling axis. We hypothesized that ALKBH3‐mediated m^1^A demethylation regulates AIM2 expression, modulating PANoptotic cell death and myocardial tissue injury. By integrating transcriptomic and epitranscriptomic profiling with molecular and functional assays, we aimed to elucidate the potential molecular mechanisms through which m^1^A influences cardiomyocyte fate during myocardial I/R. This study provides insights into the epigenetic regulation of PANoptosis and highlights m^1^A signalling as a promising avenue for therapeutic intervention in ischemic cardiomyopathy.

## 2 MATERIALS AND METHODS

### 2.1 Animals

All mice were purchased from Changsheng Biotechnology (Liaoning, China). All procedures were approved by the Ethics Committee of Harbin Medical University (approval number: IRB3026722). AIM2 floxed (AIM2^fl/fl^) and ZBED6 knock‐in (Rosa26‐LSL‐ZBED6) lines on a C57BL/6 background were generated by Cyagen Biosciences (Guangzhou, China) using CRISPR/Cas9. In brief, the following guide RNAs were used: for AIM2, gRNA‐A1 (forward strand, 5′‐GCCCTACACCTCCTAAGGTGAGG‐3′) and gRNA‐A2 (reverse strand, 5′‐CATGTCACCACACACAGCGAAGG‐3′); for ZBED6, gRNA (5′‐CTCCAGTCTTTCTAGAAGATGGG‐3′). Conditional, cardiomyocyte‐specific AIM2 knockout (Aim2^fl/fl^; Myh6‐Cre) and Cre‐dependent cardiomyocyte‐specific ZBED6 expression (Myh6‐Cre; Rosa26‐LSL‐ZBED6; referred to as ZBED6^CKI^) were generated by crossing with Myh6‐Cre mice. Genotyping primers were as follows: AIM2^fl/fl^, forward 5′‐CACACTCCTTGTCCTTAATCCTCA‐3′ and reverse 5′‐TAAAAGCAACACAGAGGTGAAGATG‐3′; Rosa26‐LSL‐Zbed6, forward 5′‐AGAAGCGCGATCACATGGTCC‐3′ and reverse 5′‐CTTTATTAGCCAGAAGTCAGATGC‐3′; Myh6‐Cre, forward 5′‐GAAATGACAGACAGATCCCTCCTATC‐3′ and reverse 5′‐CGACGATGAAGCATGTTTAGCTG‐3′. Mice were housed at 20–24°C with 40%–60% relative humidity under a 12 h light/dark cycle, with free access to standard chow and water.

### 2.2 Mouse model of myocardial I/R injury

Male mice were anaesthetized with 2% avertin (0.1 mL/10 g, intraperitoneal). After intubation, mechanical ventilation was provided (rodent ventilator R415; RWD Life Science, China; tidal volume 200 µL; 110 breaths/min). Following sterile preparation, a left thoracotomy was carried out through the third–fourth intercostal space. The left anterior descending coronary artery (LAD) was ligated 1–2 mm distal to the lower edge of the left atrium using a 7‐0 silk slipknot for 45 min, then reperfused for 24 h. Sham controls received identical surgical procedures except for LAD ligation.

### 2.3 AAV9‐mediated cardiomyocyte‐specific overexpression and knockdown of ALKBH3

Recombinant AAV9 vectors for cardiomyocyte‐specific overexpression or knockdown of ALKBH3 were constructed and packaged by GeneChem (Shanghai, China). For overexpression, the full‐length coding sequence of mouse Alkbh3 (NM_026944.2) was cloned into the GV683 backbone under the control of the cardiac troponin T (cTnT) promoter. The overexpression cassette contained a 3×FLAG tag and a T2A‐EGFP reporter, followed by WPRE and SV40 polyA signals, and was flanked by AAV2 inverted terminal repeats (ITRs). For knockdown, an RNAi sequence targeting Alkbh3 (Target ID: P25C0900; 5′‐AGCAATGTGACCGACAATTTG‐3′) or a scrambled control sequence (5′‐TTCTCCGAACGTGTCACGT‐3′) was inserted into the MIR155 scaffold within the GV683 backbone under the cTnT promoter, generating the cassette cTnT–EGFP–MIR155(shRNA)–WPRE–SV40 polyA. For in vivo gene modulation, mice received a single tail‐vein injection of AAV9‐ALKBH3 (overexpression), AAV9‐shALKBH3, or the corresponding AAV‐scramble control vectors at a dose of 1 × 10^1^ ^1^ vg per mouse in a total volume of 100 µL, 4 weeks before myocardial I/R surgery. The viral genome titer for the overexpression vector was 2.38 × 10^1^ ^3^ vg/mL, and the titer for the knockdown vector was 2.34 × 10^1^ ^3^ vg/mL. Transduction efficiency was assessed by EGFP fluorescence, and ALKBH3 overexpression/knockdown was confirmed by Western blot analysis.

### 2.4 Isolation, culture and treatment of primary mouse cardiomyocytes

Primary neonatal mouse ventricular cardiomyocytes (NMVCs) were isolated from 1–3‐day‐old pups. After surface disinfection with 75% ethanol, hearts were collected in a vertical‐flow clean bench. Tissue was placed in 0.10% trypsin‐EDTA and gently agitated at 4°C for 12 h, then minced and serially digested with 0.25% trypsin until dissociation. Cell suspensions were centrifuged (1500×*g*, 5 min) and resuspended in complete medium supplemented with 10% fetal bovine serum (FBS; Biological Industries, Israel) and 1% penicillin/streptomycin (Biosharp, China). After pre‐plating for 1 h 40 min (37°C, 5% CO_2_, 95% humidity) to reduce fibroblasts, NMVCs were collected and cultured under the same conditions for 48 h. Hypoxia/reoxygenation (H/R) was induced by hypoxia (5% CO_2_/95% N_2_, 37°C, 24 h) followed by reoxygenation (5% CO_2_/95% air, 37°C, 24 h). Where indicated, cells were transfected with siRNA or plasmids using Lipofectamine 3000 (Invitrogen, USA) or treated with inhibitors, cycloheximide (CHX) or actinomycin D (ActD). Full technical details for siRNAs, plasmids and inhibitors are provided in Table S2–S4.

### 2.5 RNA sequencing and bioinformatics analysis

Total RNA was extracted using TRIzol and treated with DNase I. RNA‐seq library were constructed using the KC Digital mRNA library prep kit (Seqhealth), incorporating 12‐nt unique molecular identifiers (UMIs) and sequenced on the DNBSEQ‐T7 platform (paired‐end 150 bp), yielding approximately 20–50 million clean reads per sample (mean chromosomal coverage >2×). Raw reads were filtered and adapter‐trimmed using fastp (v0.23.2), followed by UMI‐based deduplication and consensus sequence generation. Processed reads were aligned to the mouse reference genome GRCm39 (Ensembl release 110; Ensembl_GRCm39_110) using STAR (v2.7.6a). Gene‐ and transcript‐level read counts were obtained with featureCounts (Subread v1.5.1). Differentially expressed genes and transcripts were identified using edgeR (v3.40.2), with significance defined as Benjamini–Hochberg FDR < .05 and fold change ≥2 (|log2FC| ≥ 1).

### 2.6 m^1^A‐MeRIP‐seq and bioinformatic analysis

m^1^A‐seq was performed largely as described previously with minor modifications.^19^, ^52^ Poly(A)+ RNA (100 µg) was enriched, fragmented (70°C, 6 min) and immunoprecipitated using an anti‐m^1^A antibody coupled to Protein G magnetic beads. A fraction of both input and IP RNA was treated with alkaline buffer (50°C, 15 min) to facilitate m^1^A‐to‐m^6^A conversion for single‐base site assignment. Libraries were prepared using the EpiTM Mini LongRNA‐seq kit and sequenced on an Illumina NovaSeq 6000 platform (PE150), generating ∼40–60 million reads per library (≈10 Gb). Raw reads were quality‐checked with FastQC (v0.11.5) and trimmed using Trimmomatic (v0.36), then aligned with STAR (v2.5.2b) to the mouse reference genome GRCm38/mm10 (Ensembl annotation: Mus_musculus. GRCm38.91). PCR duplicates were removed using Picard (v2.26.10), and uniquely mapped reads were retained. m^1^A‐enriched peaks were called using MACS2 (v2.2.9.1) with matched input as control. m^1^A sites were refined by integrating peak regions with alkaline‐conversion signatures using custom scripts (±50 nt around adenosines), and metagene profiles were generated using MetaPlotR. Differential peaks were identified using DESeq2 (v1.30.1), with significance defined as Benjamini–Hochberg FDR < 0.05 and |log2FC| > 1 (fold change > 2).

### 2.7 Echocardiography

Mice were anaesthetized with 2% avertin. M‐mode echocardiography was performed (Vevo 2100; VisualSonics, Toronto, Canada; 10 MHz transducer). Left ventricular function was assessed by ejection fraction (EF), fractional shortening (FS) and left ventricular internal diameter (LVID), averaging three successive beats.

### 2.8 TTC‐Evans blue staining

After 45 min ischemia and 24 h reperfusion, the LAD was briefly re‐occluded, and 1 mL of 2% Evans Blue was injected via abdominal aorta. Hearts were excised, apex up, snap‐frozen at −80°C for 3 h, sliced transversely into four 1 mm sections, and incubated in 2% TTC (Solarbio, China) at 37°C for 10 min. Slices were imaged, and infarct size was quantified using Image‐Pro Plus.

### 2.9 Flow cytometry

Cardiomyocytes were washed twice with PBS and resuspended in 400 µL 1× binding buffer. Cell death was measured using an Annexin V‐FITC/propidium iodide (PI) kit (Abbkine, China, cat. no. E‐CK‐A211) per the manufacturer's instructions (5 µL Annexin V‐FITC + 5 µL PI; 15 min, room temperature, dark). Data were acquired on a NovoCyte flow cytometer (Agilent Technologies) and analyzed with NovoExpress. Early apoptosis was defined as Annexin V^+^/PI^−^, and late apoptosis/necrosis as Annexin V^+^/PI^+^.

### 2.10 Live/dead and TUNEL staining

Viability was assessed with a live/dead viability/cytotoxicity kit (Elabscience, China, cat. no. E‐CK‐A354). Apoptosis was evaluated with a TUNEL (terminal deoxynucleotidyl transferase dUTP nick end labelling) kit (Abbkine, cat. no. KTA2011). Live and dead cells were stained green and red; TUNEL‐positive nuclei were red, and total nuclei were counterstained blue. Fluorescence was visualized and quantified by laser scanning confocal microscopy.

### 2.11 H&E staining

Hearts were fixed in paraformaldehyde, paraffin‐embedded and sectioned. Sections were deparaffinized with xylene and rehydrated through a graded ethanol series, followed by hematoxylin and eosin (H&E) staining using a commercial kit (Solarbio, cat. no. G1120).

### 2.12 Western blotting

Heart tissue or cells were lysed in RIPA buffer with 1% protease inhibitor (Roche, Switzerland) on ice and briefly sonicated. Lysates were cleared (13 500 rpm, 15 min), supernatants collected and protein concentration determined by BCA assay (Beyotime, Shanghai, China). Proteins were separated by SDS‐PAGE (10%–12.5%) and transferred to nitrocellulose membranes. Membranes were incubated overnight at 4°C with primary antibodies (details are provided in Table S5). After washing, membranes were incubated with secondary antibodies (1:10 000; LI‐COR, USA) for 55 min at room temperature and scanned on an Odyssey infrared imaging system (LI‐COR).

### 2.13 Real‐time quantitative PCR

Total RNA was extracted from heart tissue or cardiomyocytes using TRIzol reagent (Invitrogen). RNA concentrations and purity were assessed with a NanoDrop ND‐8000 (Thermo Fisher Scientific, USA). cDNA was prepared using TransScript all‐in‐one first‐strand cDNA synthesis SuperMix (TransGen Biotech, China). qRT‐PCR (quantitative reverse transcription polymerase chain reaction) was performed with SYBR Green Master Mix (Roche, Switzerland) on an ABI 7500 system (Applied Biosystems, Foster City, CA). Relative expression was calculated by the 2^−ΔΔCt^ method.

### 2.14 Co‐immunoprecipitation

Protein A/G agarose beads (50 µL; Roche, USA) were washed with PBST (0.5% Tween‐20, pH 7.4). STAT1 (Proteintech; 50 µg/mL) or ZBED6 (Atlas Antibodies; 50 µg/mL) antibodies were pre‐bound to beads by rotating at 4°C for 2 h. Beads were pelleted by brief centrifugation and washed with PBST. Primary cardiomyocyte lysates (RIPA buffer) were then incubated with antibody‐coupled beads at 4°C for 2 h. After washing, beads were resuspended in 1× SDS‐PAGE loading buffer, heated at 95°C for 5 min, and analyzed by western blot.

### 2.15 Chromatin immunoprecipitation and promoter assay

Chromatin immunoprecipitation (ChIP) was performed using the SimpleChIP Plus Enzymatic Chromatin IP kit (Cell Signaling Technology, USA, cat. no. 9005S) to analyze the binding of ZBED6/STAT1 to the Aim2 promoter. Briefly, tissue was dissociated with a Dounce homogenizer, chromatin was enzymatically digested to 150–900 bp and cross‐linked chromatin was collected. Diluted chromatin (500 µL) was incubated with specific antibodies (anti‐ZBED6 or anti‐STAT1) and 30 µL Protein G magnetic beads at 4°C for 2 h. Normal Rabbit IgG was used as a negative immunoprecipitation control. An aliquot of chromatin (1% of total) was reserved as input DNA. Bound chromatin was eluted with 1× ChIP elution buffer (150 µL, 65°C, 30 min), purified and analyzed by qPCR using specific primers targeting the Aim2 promoter. Enrichment was quantified using the Percent Input method.

For promoter assays, STAT1‐responsive regions in the Aim2 promoter were mapped using a series of 5′‐deletion luciferase constructs. Mouse Aim2 promoter fragments (Aim2‐1, 2500 bp; Aim2‐2, 2099 bp; Aim2‐3, 566 bp; Aim2‐4, 437 bp) were amplified from genomic DNA and cloned into the pGL3‐basic luciferase vector using MluI/XhoI sites. In contrast, ZBED6‐dependent regulation was evaluated using motif‐mutant reporter constructs, in which the predicted ZBED6‐binding motifs within the Aim2 promoter were mutated by PCR‐based site‐directed mutagenesis. All constructs were verified by Sanger sequencing. Cells were co‐transfected with the indicated reporter constructs together with STAT1 or ZBED6 expression vectors (or corresponding controls) and a Renilla plasmid. Luciferase activities were measured using the Dual‐Luciferase Reporter Assay System (Promega, E1910), and firefly luciferase activity was normalized to Renilla luciferase activity. Primer sequences are provided in Table S6.

### 2.16 RNA‐binding protein immunoprecipitation

ALKBH3‐RNA‐binding protein immunoprecipitation (RIP) was performed using the Magna RIP RNA‐binding protein immunoprecipitation kit (Millipore, USA, 17–700). Fresh mouse heart tissue was lysed in RIP lysis buffer, and anti‐ALKBH3 antibody was immunoprecipitated with the kit‐supplied magnetic protein A/G beads. Bead–protein–RNA complexes were isolated magnetically, washed with RIP wash buffer and the associated RNA was purified for PCR analysis. Enrichment was quantified using the percent input method.

### 2.17 Cell counting kit‐8

Cell viability was measured using the cell counting kit‐8 (CCK‐8; Meilunbio, Dalian, China, cat. no. MA0218). Reagent was added to wells, incubated for 1.5 h at 37°C protected from light and absorbance was read at 450 nm on a microplate reader.

### 2.18 Statistical analysis

Data are presented as mean ± standard error of the mean (SEM) from at least three independent biological replicates (independent cultures or individual animals). Statistical analyses were performed in GraphPad Prism 10.0 (GraphPad Software, San Diego, CA). Two‐group comparisons used unpaired two‐tailed Student's *t*‐tests; multiple‐group comparisons used one‐way ANOVA followed by Dunnett's post hoc test. A *p*‐value < .05 was considered statistically significant. For in vivo studies, animals were allocated to groups using randomization, and investigators were blinded to group allocation during surgery and outcome assessment to minimize bias. Sample sizes were determined a priori based on a power analysis.

## 3 RESULTS

### 3.1 Cardioprotective ALKBH3 is downregulated in myocardial I/R injury in vivo

**FIGURE 1:**
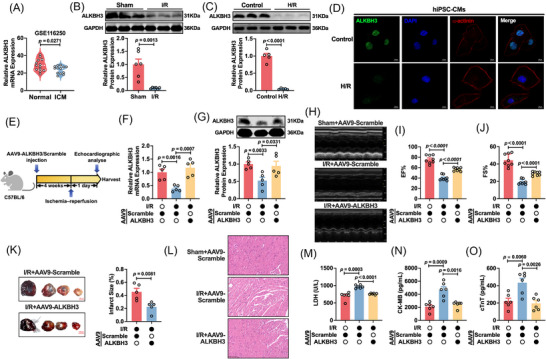
ALKBH3 overexpression limits myocardial injury during I/R in mice. (A) GEO dataset GSE116250: ALKBH3 mRNA expression in left‐ventricular tissue from patients with ICM and normal controls (*n* = 13). (B) Western blot analysis of ALKBH3 in mouse myocardium from the Sham and I/R groups (*n* = 6). (C) Western blot analysis of ALKBH3 in cardiomyocytes under normoxic or H/R conditions (*n* = 5). (D) Immunofluorescence staining—representative images and quantification of ALKBH3 fluorescence intensity—in hiPSC‐CMs under normoxic or H/R conditions (*n* = 5). Magnification: 63×. Scale bar: 10 µm. (E) Schematic of the in vivo AAV9‐mediated ALKBH3 overexpression and I/R protocols. (F, G) qRT‐PCR and western blotting confirmed myocardial ALKBH3 overexpression (*n* = 5). (H–J) Echocardiographic assessment of left‐ventricular EF and FS (n = 8). (K) Evans blue/TTC staining for infarct size quantification (*n* = 5). (L) Representative H&E‐stained myocardial sections (*n* = 3). (M–O) Serum biomarkers of injury: LDH, CK‐MB and cTnT (*n* = 6). CK‐MB, creatine kinase‐MB; cTnT, cardiac troponin T; EF, ejection fraction; FS, fractional shortening; H/R, hypoxia/reoxygenation; ICM, ischemic cardiomyopathy; I/R, ischemia/reperfusion; LDH, lactate dehydrogenase; qRT‐PCR, quantitative reverse‐transcription PCR.

Given that ALKBH3's role in myocardial I/R injury is unclear and that ALKBH3 is a well‐characterized demethylase that removes m^1^A from mRNA, we first examined the ALKBH3 expression dynamics during I/R. Publicly available transcriptomic datasets (GEO accession: GSE116250) showed significantly reduced ALKBH3 mRNA levels in myocardial samples from patients with ischemic cardiomyopathy compared with those from healthy controls, implicating ALKBH3 in the pathology of ischemic cardiomyopathy (Figure 1A). Consistently, in a murine I/R model, ALKBH3 protein levels were decreased in myocardial tissue relative to the sham group, as shown by western blotting (Figure 1B), and ALKBH3 expression was likewise reduced in cardiomyocytes subjected to hypoxia/reoxygenation (H/R) (Figure 1C). To further validate these findings in a human‐relevant model, we performed immunofluorescence staining of human‐induced pluripotent stem cell‐derived cardiomyocytes (hiPSC‐CMs), which similarly exhibited a marked reduction in ALKBH3 protein levels following H/R challenge (Figure 1D). To assess their functional relevance, we generated an cardiomyocyte‐targeted AAV9‐ALKBH3 overexpression model in vivo. Mice that received AAV9‐ALKBH3 or control AAV underwent I/R 4 weeks later and were analyzed 24 h post‐injury (Figure 1E). qRT‐PCR and western blotting confirmed the robust cardiac overexpression of ALKBH3 (Figure 1F,G). Echocardiography demonstrated improved left‐ventricular function in ALKBH3‐overexpressing mice after I/R, with a higher ejection fraction and fractional shortening than those in the control mice (Figure 1H–J). Evans blue/TTC staining revealed a smaller infarct size in the ALKBH3 group (Figure 1K). Histological analysis (H&E) revealed attenuation of I/R‐induced structural disruption and inflammatory infiltration following ALKBH3 overexpression (Figure 1L). Serum injury markers (LDH, CK‐MB and cTnT) were elevated after I/R but were significantly reduced in AAV9‐ALKBH3–treated mice (Figure 1M–O). Together, these findings indicate that ALKBH3 is downregulated in myocardial I/R and that restoring ALKBH3 expression confers cardioprotection by preserving cardiac function and limiting myocardial injury.

### 3.2 ALKBH3 exerts a protective effect against cardiomyocyte death

**FIGURE 2:**
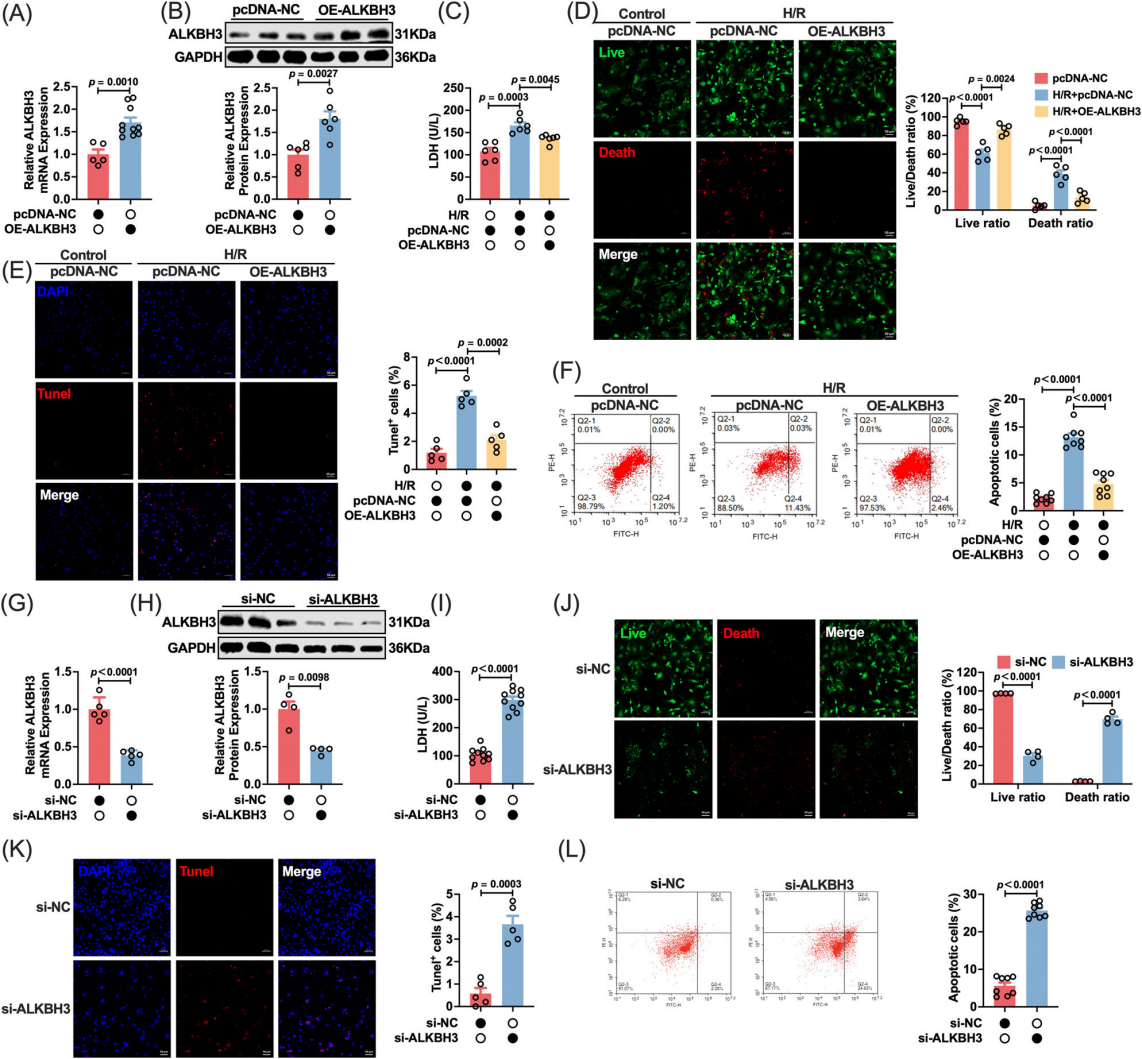
ALKBH3 confers cytoprotection to cardiomyocytes during H/R stress. (A) qRT‐PCR analysis of ALKBH3 mRNA in cardiomyocytes transfected with NC or ALKBH3 overexpression plasmids (*n* = 5). (B) Western blot analysis confirming ALKBH3 overexpression (*n* = 6). (C) LDH release in H/R‐treated cardiomyocytes with or without ALKBH3 overexpression (*n* = 6). (D) Live/dead staining—representative images and quantification of viable versus dead cells—in H/R groups with or without ALKBH3 overexpression (*n* = 5). Magnification: 10×. Scale bar: 50 µm. (E) TUNEL staining and (F) Annexin V/PI flow cytometry showing apoptosis in the indicated groups (*n* = 4). Magnification: 10×. Scale bar: 50 µm. (G, H) qRT‐PCR and western blot analysis of ALKBH3 expression after siRNA‐mediated knockdown (si‐ALKBH3) versus NC siRNA (si‐NC) (*n* = 4, 5). (I) LDH release in the si‐NC and si‐ALKBH3 groups (*n* = 6). (J) Live/Dead staining—representative images and quantification—and (K) TUNEL staining after ALKBH3 knockdown (*n* = 4). Magnification: 10×. Scale bar: 50 µm. (L) Annexin V/PI flow cytometry analysis of apoptosis after ALKBH3 knockdown (*n* = 8). H/R, hypoxia/reoxygenation; LDH, lactate dehydrogenase; NC, negative control; PI, propidium iodide; qRT‐PCR, quantitative reverse‐transcription PCR.

Building on our in vivo observations, we used an in vitro H/R model to isolate cell‐autonomous effects and minimize confounding in vivo factors. We constructed an overexpression model to assess its functional relevance. ALKBH3 overexpression was confirmed using western blotting and qRT‐PCR (Figure 2A,B). Consistent with the in vivo results, ALKBH3 overexpression alleviated H/R‐induced injury, as evidenced by reduced LDH release (Figure 2C) and improved cell viability in live/dead assays. Quantification showed that H/R treatment reduced cell viability and increased cell death, which was reversed by ALKBH3 overexpression (Figure 2D). TUNEL staining further demonstrated reduced H/R‐induced cell death with ALKBH3 overexpression (Figure 2E), as confirmed by flow cytometry (Figure 2F).

Given these findings, we explored whether knockdown of ALKBH3 alone could result in cardiomyocyte damage. Conversely, siRNA‐mediated knockdown efficiently reduced ALKBH3 at the mRNA and protein levels (Figure 2G, H) and recapitulated the H/R‐induced injury phenotype, as indicated by increased LDH release (Figure 2I), increased cell death in live/dead assays (Figure 2J), an increased number of TUNEL‐positive cells (Figure 2K) and elevated apoptosis by flow cytometry (Figure 2L). Collectively, these data demonstrate that ALKBH3 is a critical determinant of cardiomyocyte survival under H/R stress; its overexpression mitigates cell injury and death, whereas its knockdown aggravates these effects.

### 3.3 ALKBH3 suppresses cardiomyocyte PANoptosis during myocardial I/R injury

**FIGURE 3:**
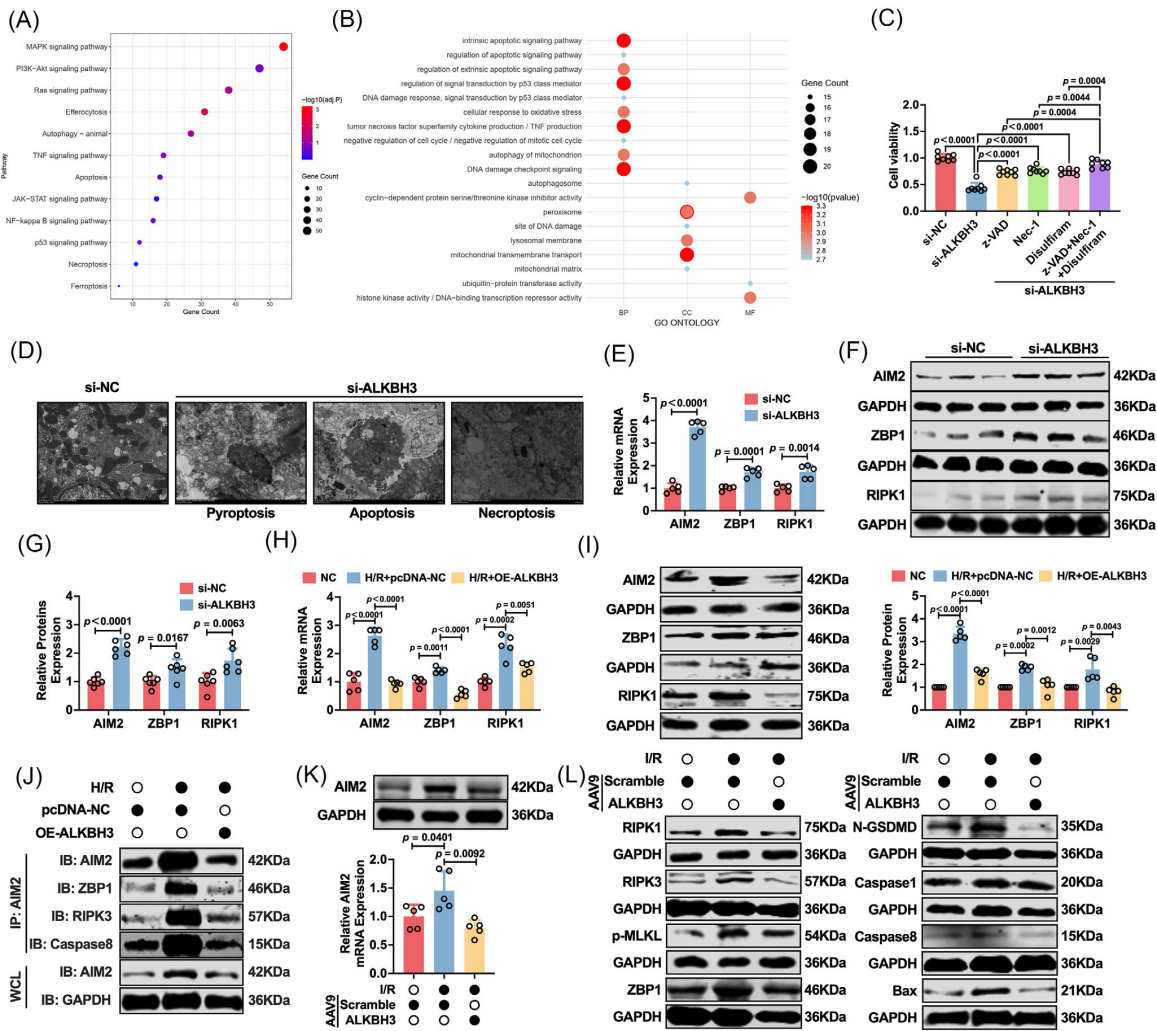
ALKBH3 suppresses PANoptosis during myocardial I/R injury. (A, B) KEGG and GO enrichment of differentially expressed genes from RNA‐seq after ALKBH3 knockdown, highlighting pathways related to apoptosis, necroptosis and inflammation. (C) Cell viability following ALKBH3 knockdown and treatment with individual or combined pathway inhibitors (z‐VAD, Nec‐1, disulfiram) (*n* = 6). (D) Transmission electron microscopy showing ultrastructural features consistent with PANoptosis after ALKBH3 silencing. (E–G) qRT‐PCR and western blot analysis of PANoptosis receptors (AIM2, ZBP1, RIPK1) upon ALKBH3 knockdown (*n* = 5). (H, I) qRT‐PCR and western blot analysis of PANoptosis receptors in H/R groups with or without ALKBH3 overexpression (*n* = 5). (J) Co‐IP demonstrating the interaction between AIM2 and PANoptosome components (ZBP1, RIPK3 and caspase‐8) under H/R conditions with or without ALKBH3 overexpression (*n* = 3). (K) Western blot analysis of AIM2 in mouse myocardial tissue (*n* = 5). (L) Western blot analysis of PANoptosis‐associated proteins (RIPK1, RIPK3, p‐MLKL, ZBP1, N‐GSDMD, caspase‐1, caspase‐8, Bax) (*n* = 5). Co‐IP, co‐immunoprecipitation; GO, Gene Ontology; H/R, hypoxia/reoxygenation; I/R, ischemia/reperfusion; KEGG, Kyoto Encyclopedia of Genes and Genomes; qRT‐PCR, quantitative reverse‐transcription PCR.

To determine the mechanisms underlying the cardioprotective effects of ALKBH3, we first performed transcriptome‐wide RNA sequencing after ALKBH3 knockdown in cardiomyocytes. Differential expression analysis revealed extensive remodelling of the transcriptional landscape, with significant enrichment of pathways involved in inflammation and multiple programmed cell death modalities, including apoptosis, necroptosis and ferroptosis (Figure 3A,B). Gene Ontology and Kyoto Encyclopedia of Genes and Genomes analyses likewise highlighted inflammatory signalling and regulated cell‐death pathways. Given the interplay between inflammation and cell death and our previous work implicating PANoptosis,^53^, ^54^, ^55^ an inflammatory programmed cell death program integrating pyroptosis, apoptosis and necroptosis, we investigated whether PANoptosis functions downstream of ALKBH3 deficiency.

Cell viability assays revealed a marked reduction in cell viability following ALKBH3 knockdown. While pharmacological blockade of individual distinct death arms using z‐VAD (to inhibit caspase‐dependent apoptosis), Nec‐1 (to inhibit RIPK1‐dependent necroptosis) or disulfiram (to inhibit GSDMD‐mediated pyroptosis) conferred only partial protection, the combined inhibition of all three pathways resulted in a significantly more robust rescue of cell viability compared with any single agent alone (Figure 3C). To corroborate these pharmacological findings using a genetic approach, we used specific small interfering RNAs (siRNAs) targeting the key effectors caspase‐1, GSDMD, RIPK3 and MLKL (Figure S1A). Consistent with the inhibitor data, silencing of individual components conferred partial protection against cell death induced by ALKBH3 deficiency. The concurrent knockdown of all four effectors resulted in a significantly more pronounced restoration of cell viability than single‐gene silencing, mirroring the synergistic effect observed with the inhibitor cocktail (Figure S1B). Transmission electron microscopy further revealed ultrastructural features consistent with pyroptosis, apoptosis and necroptosis, including mitochondrial swelling, chromatin condensation and plasma membrane disruption in ALKBH3‐deficient cells (Figure 3D). Collectively, these findings suggest that ALKBH3 acts as a suppressor of PANoptosis.

AIM2, ZBP1 and RIPK1 were prioritized as candidate PANoptosis sensors/mediators.^56^, ^57^, ^58^ qRT‐PCR and western blotting confirmed that AIM2 exhibited the most robust and sustained upregulation upon ALKBH3 silencing, whereas ZBP1 and RIPK1 showed minimal changes (Figure 3E–G). Under hypoxia/reoxygenation (H/R) conditions, AIM2 was induced and showed a far greater fold increase than ZBP1 or RIPK1. ALKBH3 overexpression effectively blunted H/R‐induced AIM2 upregulation, with a lesser effect on ZBP1 or RIPK1 (Figure 3H,I). To determine whether these changes in expression changes translated into functional PANoptosome assembly, we performed co‐immunoprecipitation (co‐IP) assays using an anti‐AIM2 antibody. In H/R‐treated cardiomyocytes, we observed a markedly enhanced interaction between AIM2 and key PANoptosis components, including ZBP1, RIPK3 and caspase‐8, indicating enhanced complex formation. However, this interaction was significantly disrupted by ALKBH3 overexpression (Figure 3J). To validate this regulatory relationship in a human context, we examined ALKBH3 and AIM2 protein levels in AC16 cardiomyocytes. Consistent with murine data, H/R challenge robustly upregulated AIM2 expression in AC16 cells, and this effect was markedly reversed by ALKBH3 overexpression (Figure S2A). In myocardial tissue, ALKBH3 overexpression reduced AIM2 and key PANoptosis markers, including RIPK1, RIPK3, p‐MLKL, ZBP1, caspase‐1, caspase‐8, N‐GSDMD and Bax, as shown by western blotting, further supporting the inhibition of PANoptosis (Figure 3K,L; Figure S2B). These data indicate that PANoptosis is a critical downstream mediator of ALKBH3‐dependent control of cardiomyocyte death under ischemic stress, with AIM2 emerging as a key regulatory target through which ALKBH3 modulates this cell death program.

### 3.4 AIM2 is a critical mediator of the anti‐PANoptosis effect of ALKBH3

AIM2, a cytosolic DNA sensor and inflammasome component, is also a key initiator of PANoptosis through the assembly of the AIM2‐PANoptosome complex,^59^, ^60^, ^61^ providing a scaffold for coordinated pyroptotic, apoptotic and necroptotic execution.

To establish the role of AIM2 in myocardial I/R injury independent of ALKBH3, we generated cardiomyocyte‐specific AIM2 knockout mice (AIM2^fl/fl^Myh6^Cre^) and subjected them to I/R. Compared with AIM2^fl/fl^ controls, AIM2^fl/fl^Myh6^Cre^ mice exhibited markedly reduced myocardial injury and improved cardiac function, with lower levels of serum injury markers, supporting the deleterious role of AIM2 in I/R (Figure S3).

**FIGURE 4:**
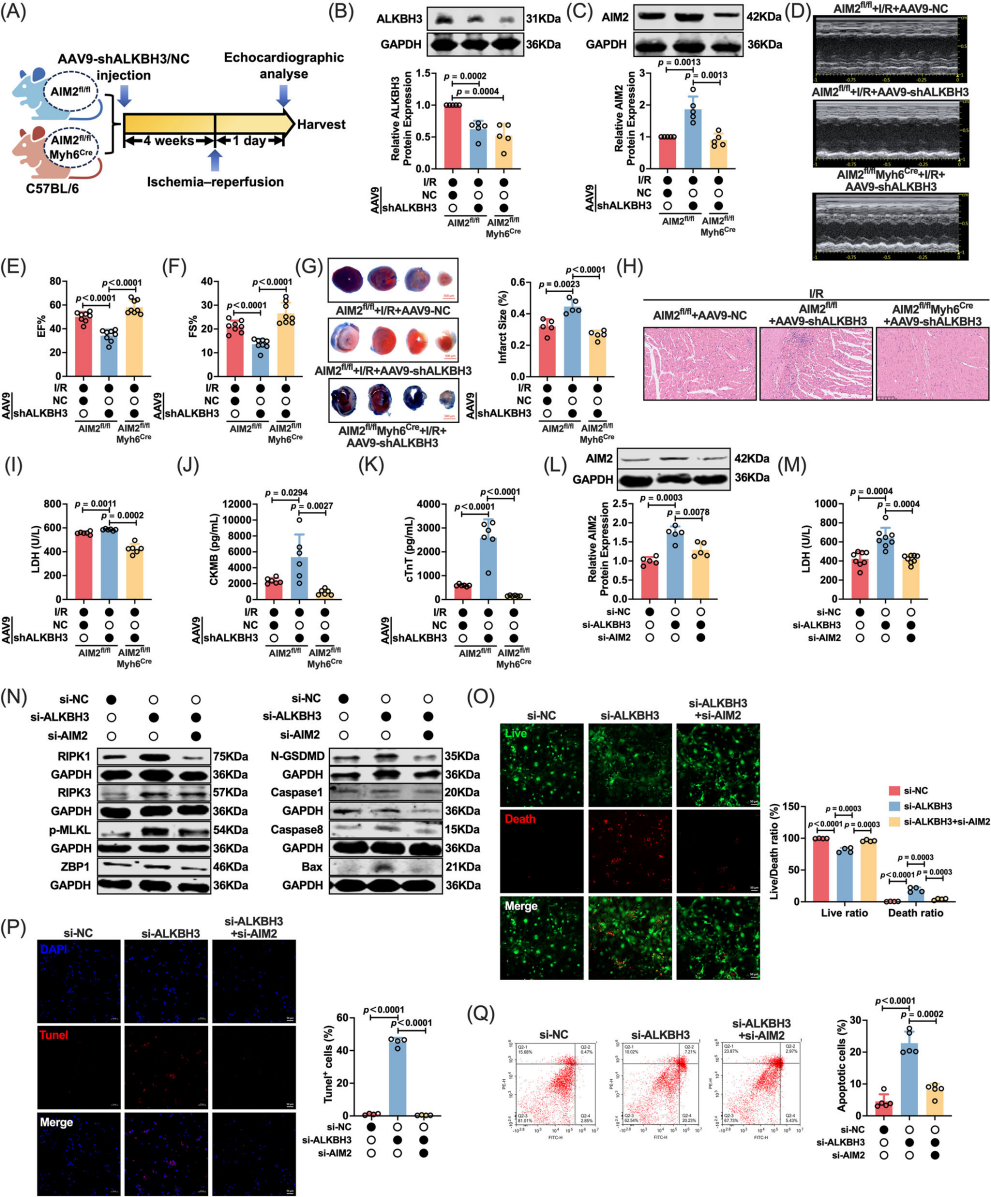
AIM2 mediates ALKBH3‐regulated PANoptosis and myocardial injury in vitro and in vivo. (A) Experimental schema: AIM2^fl/fl^ (Myh6^Cre−^) and cardiomyocyte‐specific AIM2 knockout mice (AIM2^fl/fl^Myh6^Cre−^) received tail‐vein AAV9 carrying control shRNA (AAV9‐NC) or ALKBH3 shRNA (AAV9‐shALKBH3). I/R surgery and tissue collection were performed at the indicated time points. (B, C) Western blot analysis of ALKBH3 and AIM2 in mouse myocardium (*n* = 5). (D–F) Representative echocardiograms and quantification of left‐ventricular EF and FS (*n* = 8). (G) Evans blue/TTC staining and infarct size quantification (*n* = 5). (H) Representative H&E‐stained myocardial sections (*n* = 3). (I–K) Serum LDH, CK‐MB and cTnT levels (*n* = 6). (L) Western blot analysis of AIM2 expression after knockdown of ALKBH3 and/or AIM2 (*n* = 5). (M) LDH release with the indicated knockdown (*n* = 8). (N) Western blots of PANoptosis‐associated proteins (RIPK1, RIPK3, p‐MLKL, ZBP1, N‐GSDMD, caspase‐1, caspase‐8 and Bax) (*n* = 5). (O) Live/dead staining and quantification (*n* = 4). Magnification: 10×. Scale bar: 50 µm. (P) TUNEL staining and quantification (*n* = 4). Magnification: 10×. Scale bar: 50 µm. (Q) Annexin V/PI flow cytometry analysis of apoptosis (*n* = 5). CK‐MB, creatine kinase‐MB; cTnT, cardiac troponin T; EF, ejection fraction; FS, fractional shortening; I/R, ischemia/reperfusion; LDH, lactate dehydrogenase; PI, propidium iodide.

Next, we tested whether AIM2 mediates the effects of ALKBH3 deficiency in vivo using AAV9‐mediated cardiomyocyte‐targeted ALKBH3 knockdown (AAV9‐shALKBH3) (Figure S4A). Mice were assigned to three groups: AIM2^fl/fl^ + AAV9‐NC, AIM2^fl/fl^ + AAV9‐shALKBH3 and AIM2^fl/fl^Myh6^Cre^ + AAV9‐shALKBH3 (Figure 4A). Western blotting confirmed a substantial reduction in AIM2 protein levels in AIM2^fl/fl^Myh6^Cre^ hearts and effective ALKBH3 knockdown after AAV9‐shALKBH3 (Figure 4B,C). Echocardiography revealed that ALKBH3 knockdown worsened cardiac performance [decreased ejection fraction (EF) and fractional shortening (FS)] in AIM2^fl/fl^ mice, whereas AIM2 deficiency partially rescued this dysfunction (Figure 4D–F). Consistently, the infarct size (Evans blue/TTC) increased with ALKBH3 knockdown and was markedly attenuated by AIM2 deletion (Figure 4G). Histological analysis revealed aggravated structural injury after ALKBH3 silencing, which was ameliorated in the AIM2^fl/fl^Myh6^Cre^ background (Figure 4H). Serum LDH, CK‐MB and cTnT levels increased following ALKBH3 knockdown and were partially normalized by AIM2 deletion (Figure 4I–K).

To evaluate the cell‐autonomous effects, we silenced ALKBH3 alone or in combination with AIM2 in cardiomyocytes. Knockdown of AIM2 was validated by qRT‐PCR (Figure S4B). Western blot analysis showed that AIM2 co‐silencing counteracted the ALKBH3 knockdown‐induced increase in AIM2 protein levels (Figure 4L). Functionally, AIM2 knockdown blunted the increase in LDH release induced by ALKBH3 silencing (Figure 4M). ALKBH3 knockdown activated PANoptosis‐associated markers (RIPK1, RIPK3, p‐MLKL, ZBP1, N‐GSDMD, caspase‐1, caspase‐8 and Bax), whereas co‐silencing with AIM2 significantly attenuated these changes (Figure 4N; Figure S4C). Live/dead cell staining revealed increased cell death with si‐ALKBH3, which was partially reversed by si‐AIM2 (Figure 4O). TUNEL staining revealed more apoptotic cells in the si‐ALKBH3 group, which was mitigated by AIM2 co‐knockdown (Figure 4P). Flow cytometry results further corroborated these findings (Figure 4Q).

Together, these data indicate that ALKBH3 inhibits cardiomyocyte PANoptosis primarily by limiting AIM2 expression, and that the ALKBH3/AIM2 axis is a critical determinant of cell death and cardiac dysfunction during myocardial I/R injury.

### 3.5 ALKBH3 suppresses AIM2 expression via m^1^A demethylation of ZBED6

**FIGURE 5:**
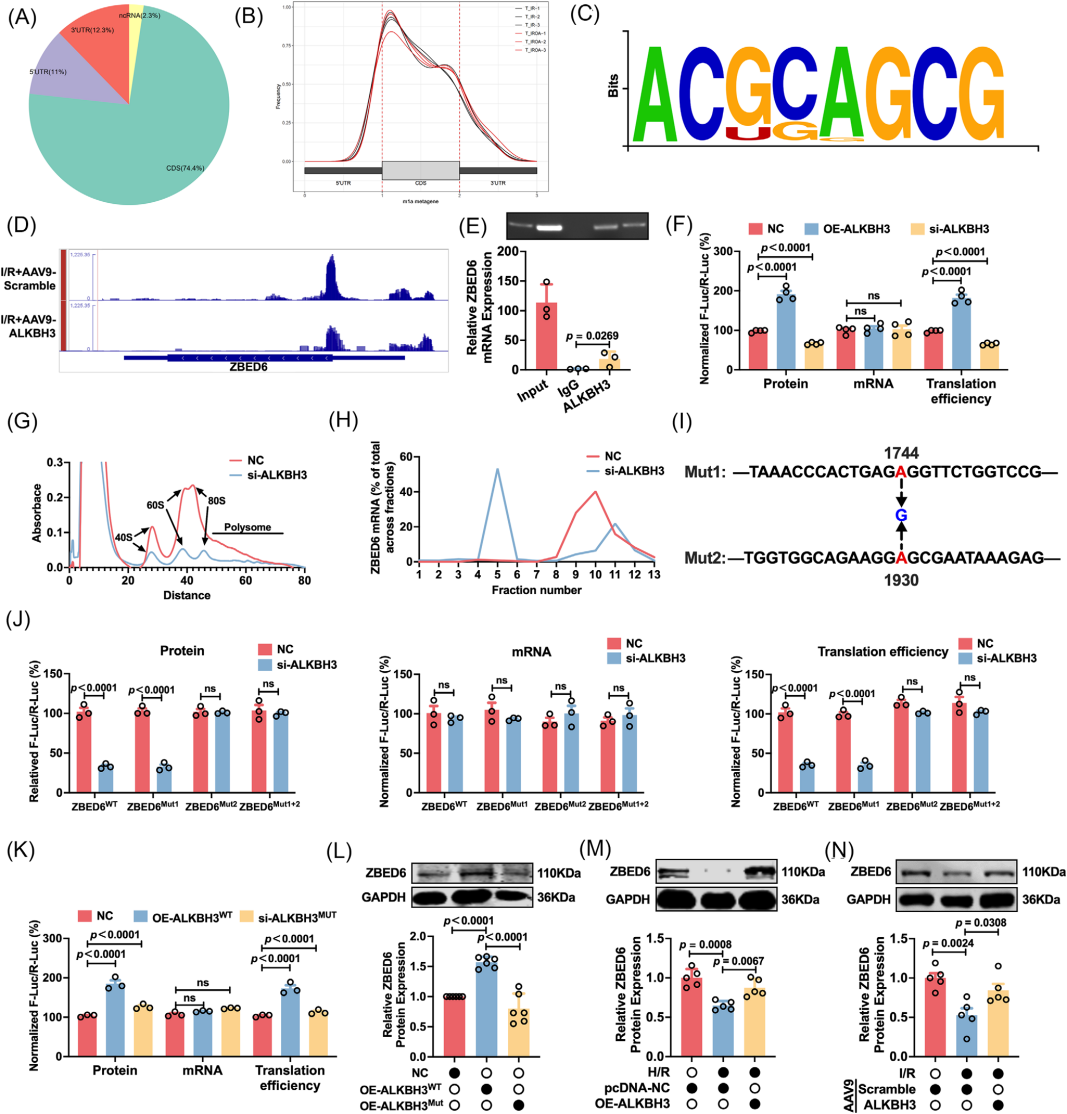
ALKBH3 regulates AIM2 expression through m^1^A‐mediated translational control of ZBED6. (A, B) Distribution of m^1^A peaks across transcript regions by m^1^A MeRIP‐seq in I/R hearts with or without ALKBH3 overexpression. (C) Motif enrichment analysis of sequences flanking the m^1^A peaks. (D) MeRIP‐seq signal tracks showing decreased m^1^A on ZBED6 mRNA near the CDS–5′UTR junction after ALKBH3 overexpression. (E) RIP assay confirming ALKBH3 binding to ZBED6 mRNA (*n* = 3). (F) Dual‐luciferase reporter assay showing reduced translational efficiency of ZBED6 after ALKBH3 knockdown (*n* = 4). (G, H) Polysome profiling analysis showing the distribution of ZBED6 mRNA across sub‐polysome, light polysome and heavy polysome fractions. Note the shift of ZBED6 transcripts from heavy polysomes to non‐translating fractions upon ALKBH3 silencing (*n* = 3). (I, J) Identification of functional m^1^A sites. Relative luciferase activity of wild‐type (WT) and point‐mutant (A1744G and A1930G) ZBED6 reporters in cardiomyocytes upon ALKBH3 knockdown (*n* = 3). (K) Relative luciferase activity of the ZBED6 reporter in cardiomyocytes overexpressing vector, wild‐type (WT) ALKBH3 or the catalytically inactive mutant (Mut) (*n* = 3). (L) Western blot analysis of ZBED6 protein levels in cardiomyocytes overexpressing Vector, WT ALKBH3, or catalytically inactive mutant (*n* = 5). (M) Western blot analysis of ZBED6 protein in H/R‐treated cardiomyocytes with ALKBH3 overexpression (n = 5). (N) Western blot analysis of ZBED6 protein in I/R hearts with ALKBH3 overexpression (*n* = 5). CDS, coding sequence; H/R, hypoxia/reoxygenation; I/R, ischemia/reperfusion; MeRIP‐seq, methylated RNA immunoprecipitation sequencing; RIP, RNA immunoprecipitation; UTR, untranslated region; WT, wild‐type.

Having identified AIM2 as a key downstream effector of ALKBH3‐regulated PANoptosis, we investigated its underlying mechanism. As ALKBH3 functions as an m^1^A demethylase, we hypothesized that it modulates target gene expression through m^1^A removal. Transcriptome‐wide m^1^A MeRIP‐seq was performed on I/R hearts overexpressing ALKBH3. The data showed that ALKBH3 markedly reshaped the myocardial m^1^A landscape, with a global decrease in m^1^A peaks consistent with demethylase activity. Altered peaks were enriched in coding sequences (CDS; 74.4%), with the most pronounced changes near the 5′ end of the CDS, suggesting preferential modulation at the translation initiation proximal sites (Figure 5A,B). Motif enrichment analysis was also performed (Figure 5C). AIM2 mRNA displayed no detectable m^1^A peaks, indicating that ALKBH3 does not regulate AIM2 directly via m^1^A demethylation.

Therefore, we hypothesized that ALKBH3 demethylates an upstream transcription factor (TF) that controls AIM2. Integrating differentially m^1^A‐modified genes with TFs annotated for AIM2 regulation (AnimalTFDB 3.0), we identified ZBED6, a transcriptional repressor previously characterized by our group,^62^ as a candidate epitranscriptomic effector. Peak visualization confirmed a marked reduction in m^1^A on ZBED6 in ALKBH3‐overexpressing samples, with peaks clustered near the CDS–5′UTR junction, a region that influences translation efficiency (Figure 5D). RIP assays demonstrated the direct binding of ALKBH3 to ZBED6 mRNA (Figure 5E), supporting a targeted post‐transcriptional mechanism.

Given the region‐specific effects of m^1^A on mRNA metabolism, we investigated how ZBED6 translation was affected. Prior work has indicated that m^1^A near the CDS–5′UTR junction can impede ribosome elongation, reducing translation efficiency and fidelity,^19^, ^57^ whereas m^1^A enrichment within the 5′UTR has been associated with enhanced mRNA stability.^55^, ^56^, ^58^ Actinomycin D and cycloheximide chase experiments revealed no significant changes in ZBED6 mRNA stability or protein degradation following ALKBH3 knockdown (Figure S5A,B), indicating that translational control was the primary mechanism. Consistent with this, dual‐luciferase reporters fusing the ZBED6 CDS to firefly luciferase (pmiR‐GLO) showed a significantly reduced translation efficiency upon ALKBH3 knockdown (Figure 5F), indicating that m^1^A negatively regulates ZBED6 translation. To corroborate these findings with endogenous evidence, we performed polysome profiling analysis. The global polysome profile revealed that ALKBH3 silencing led to a dramatic reduction in 60S, 80S and polysome fractions, indicating a broad suppression of global translation in cardiomyocytes (Figure 5G). We next assessed the specific translation efficiency of ZBED6 by measuring its mRNA distribution across the fractions. As shown in Figure 5H, ZBED6 transcripts were highly enriched in the heavy polysome fractions (fractions 9–11), indicating robust active translation. However, ALKBH3 silencing caused a significant shift in ZBED6 mRNA expression from heavy polysomes to non‐translating sub‐polysome/monosome fractions (fraction 5), directly confirming that ALKBH3 is required for efficient ZBED6 translation efficiency. To identify the functional m^1^A site responsible for this regulation, we used FIMO (v5.5.9) to scan m^1^A MeRIP‐seq‐enriched peaks on ZBED6 mRNA and predicted putative m^1^A motif sites. We then generated luciferase reporters carrying point mutations in the candidate motifs (A1744G and A1930G) of the ZBED6 gene (Figure 5I). In cells transfected with si‐ALKBH3, translational suppression observed with the wild‐type and A1744G reporters was abolished in the A1930G mutant, indicating that A1930 is the critical site that governing translational efficiency, which is located within the CDS region (Figure 5J). We further verified the requirement for ALKBH3 enzymatic activity using catalytically inactive mutants (H191A/D193A/H257A).^63^ Luciferase assays showed that, although wild‐type ALKBH3 overexpression robustly enhanced ZBED6 translation, the catalytically dead mutant did not (Figure 5K). Consistent with this, western blot analysis confirmed that wild‐type ALKBH3, but not the catalytic mutant, significantly increased ZBED6 protein levels (Figure 5L). Consistent with the loss of ALKBH3, decreased ZBED6 protein levels were observed in the I/R myocardium and H/R‐treated cardiomyocytes (Figure S6A,B). ALKBH3 overexpression effectively reversed this effect (Figure 5M,N). We further validated this regulatory conservation in human AC16 cardiomyocytes, in which ALKBH3 silencing significantly reduced ZBED6 levels, whereas ALKBH3 overexpression robustly rescued ZBED6 expression under H/R conditions (Figure S6C).

To validate the regulatory relationship between ZBED6 and AIM2, we examined whether ZBED6 directly affects AIM2 expression in cardiomyocytes. ZBED6 overexpression significantly reduced AIM2 mRNA levels (Figure S6D,E), and western blotting further confirmed that ZBED6 overexpression markedly suppressed AIM2 protein expression under H/R conditions (Figure S6F). These findings demonstrate that ZBED6 negatively regulates AIM2 expression, implicating ZBED6 as a potential upstream transcriptional repressor of AIM2. To further explore the protective role of ZBED6 against I/R injury in vivo, we established cardiomyocyte‐specific ZBED6 conditional knock‐in mice (ZBED6^CKI^) and subjected them to I/R (Figure S7A–C). ZBED6^CKI^ mice exhibited significantly improved cardiac function compared with the Rosa26‐LSL‐ZBED6 (Myh6^Cre−^; referred to as ZBED6^Ctrl^)+I/R mice, as evidenced by the increased EF and FS (Figure S7D–F). Consistently, serum LDH, CK‐MB and cTnT levels were significantly decreased in ZBED6^CKI^ mice following I/R, indicating alleviation of myocardial injury (Figure S7G–I). To determine the anti‐PANoptotic role of ZBED6, we examined the effects of ZBED6 knockdown on cardiomyocyte viability. As shown in the viability assay, the knockdown of ZBED6 significantly decreased cell survival compared with that in the negative control (NC) group. Although treatment with individual inhibitors (z‐VAD, Nec‐1 or disulfiram) partially rescued the viability of si‐ZBED6‐transfected cells, the combined administration of all three inhibitors resulted in a significantly more robust recovery of cell viability (Figure S8A–C). These results confirmed that ZBED6 deficiency reproduces the PANoptotic phenotype observed in ALKBH3‐deficient cells.

Collectively, these findings indicate that ALKBH3 directly enhances ZBED6 translation by demethylating m^1^A on ZBED6 mRNA, and that ZBED6 suppresses AIM2 expression. Thus, ZBED6 serves as a mechanistic bridge linking ALKBH3 to AIM2 transcriptional control and PANoptosis regulation.

### 3.6 ZBED6 restrains AIM2 indirectly by promoting STAT1 ubiquitination and degradation

**FIGURE 6:**
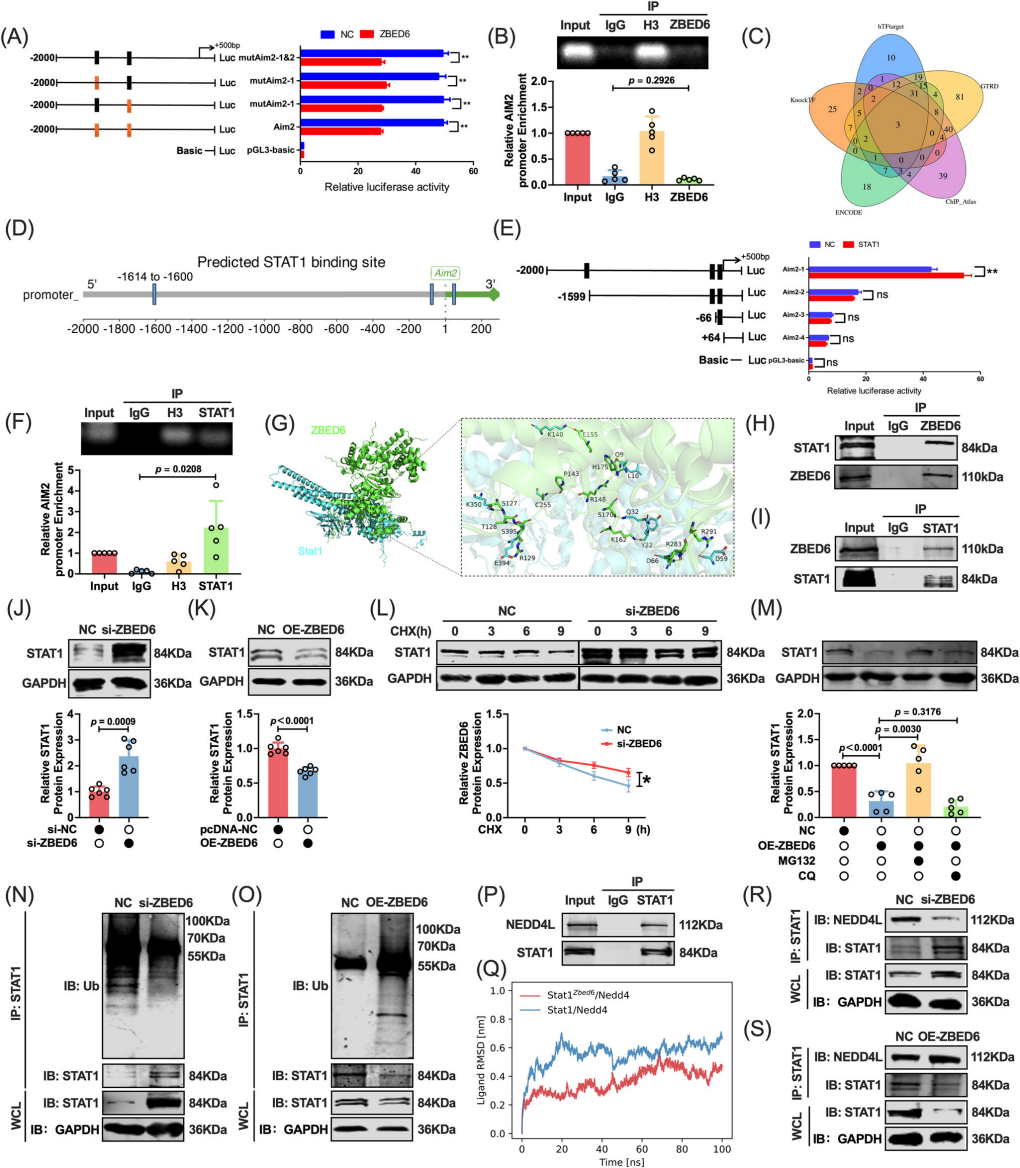
ZBED6 regulates AIM2 expression via STAT1‐dependent transcriptional activation. (A) Dual‐luciferase assay to assess the effect of ZBED6 on AIM2 promoter activity. (B) ChIP‐qPCR analysis of ZBED6 occupancy at the AIM2 promoter region (*n* = 5). (C) Venn diagram showing transcription‐factor overlap across five databases (KnockTF, ENCODE, ChIP‐Atlas, hTFtarget and GTRD). (D) Predicted STAT1‐binding sites within the AIM2 promoter identified using JASPAR database. (E) Dual‐luciferase assay to test the effect of STAT1 on AIM2 promoter activity. (F) ChIP‐qPCR confirming STAT1 enrichment at the AIM2 promoter (*n* = 5). (G) Molecular docking model of the ZBED6–STAT1 interaction. (H, I) Co‐IP demonstrating ZBED6‐STAT1 interaction in cardiomyocytes (*n* = 3). (J, K) Western blots showing STAT1 levels after ZBED6 knockdown or overexpression (*n* = 5). (L) CHX chase assay assessing STAT1 protein stability following ZBED6 knockdown (*n* = 5). (M) Western blotting of STAT1 in ZBED6‐overexpressing cells treated with MG132 (proteasome inhibitor) or chloroquine (CQ; lysosomal inhibitor) (*n* = 5). (N, O) Co‐IP ubiquitination assay showing the effects of ZBED6 knockdown and overexpression on STAT1 ubiquitination (*n* = 4). (P) Co‐IP demonstrating STAT1–NEDD4L interaction in cardiomyocytes (*n* = 3). (Q) Structural modeling of the ZBED6–STAT1–NEDD4L ternary complex derived from MD simulations, accompanied by RMSD plots and binding free energy calculations (MM‐GBSA) analyzing the interaction affinity of NEDD4L with STAT1 in the presence or absence of ZBED6. (R, S) Co‐IP analysis examining the recruitment of NEDD4L to STAT1 in cardiomyocytes with ZBED6 overexpression or knockdown (*n* = 3). (R, S) qRT‐PCR and western blot analysis of AIM2 expression after knockdown of ZBED6 and STAT1 (*n* = 3). ^*^ *P* < 0.05 vs NC. CHX, cycloheximide; ChIP‐qPCR, chromatin immunoprecipitation qPCR; Co‐IP, co‐immunoprecipitation; CQ, chloroquine; NC, negative controlqRT‐PCR, quantitative reverse‐transcription PCR.

To determine the mechanism by which ZBED6 regulates AIM2 transcription, we performed AIM2 promoter luciferase assays using a series of promoter‐mutated plasmids. ZBED6 overexpression significantly suppressed AIM2 promoter activity, indicating its role in AIM2 transcriptional control. However, mutation of the putative ZBED6‐binding sites did not alter promoter activity, and ChIP‐qPCR showed no ZBED6 enrichment in the AIM2 promoter (Figure 6A,B). These results suggest that ZBED6 indirectly influences AIM2 expression, possibly through another TF that drives AIM2 expression.

Next, we intersected the TF annotations across the five databases (KnockTF, ENCODE, ChIP‐Atlas, hTFtarget and GTRD). The genes STAT1, STAT3 and MYC overlapped across all five datasets (Figure 6C; Table S7). Protein–protein interaction prediction prioritized STAT1 (signal transducer and activator of transcription 1) as a potential ZBED6 interactor linking ZBED6 to AIM2 regulation. Using the JASPAR database, we identified three candidate STAT1 sites within the 2‐kb Aim2 promoter (Figure 6D). Consistent with direct activation, STAT1 overexpression enhanced AIM2 promoter activity, whereas truncation of the predicted STAT1 site (‐1614 to ‐1600 bp) markedly reduced the promoter activity (Figure 6E). ChIP‐qPCR confirmed STAT1 enrichment in the AIM2 promoter region (Figure 6F), supporting the hypothesis that STAT1 is a direct AIM2 transcriptional activator.

We investigated the relationship between ZBED6 and STAT1 expression. Molecular docking predicted a stable ZBED6–STAT1 interface involving several residues (Figure 6G). Co‐IP confirmed these physical interactions in cardiomyocytes (Figure 6H,I). Mechanistically, ZBED6 depletion increased STAT1 protein abundance, whereas ZBED6 overexpression decreased it (Figure 6J,K). CHX chase assays showed prolonged STAT1 half‐life upon ZBED6 knockdown (Figure 6L). In ZBED6‐overexpressing cells, the proteasome inhibitor MG132, but not the lysosomal inhibitor chloroquine (CQ), restored STAT1 levels (Figure 6M), indicating proteasome‐dependent turnover. Ubiquitination assays revealed increased STAT1 ubiquitination upon ZBED6 overexpression and reduced ubiquitination upon ZBED6 knockdown (Figure 6N,O), indicating that ZBED6 promotes STAT1 degradation via the ubiquitin‐proteasome pathway. However, as ZBED6 lacks an intrinsic catalytic domain for ubiquitination, we hypothesized that it promotes the interaction between STAT1 and a specific E3 ligase. UbiBrowser prediction coupled with co‐IP screening prioritized NEDD4L as the primary effector for further investigation, as it exhibited the most robust physical interaction with STAT1 (Figure S9A; Figure 6P). Molecular docking analysis confirmed a stable binding interface between NEDD4L and STAT1 (Figure S9B). To elucidate the regulatory role of ZBED6 in this interaction, we performed molecular dynamics (MD) simulations. The results demonstrated that the ZBED6–STAT1 binary complex exhibited a much stronger binding capability for NEDD4L than STAT1 alone, suggesting that ZBED6 acts as a molecular scaffold to stabilize the ternary interactions (Figure 6Q). We experimentally validated the function of this scaffold in cardiomyocytes by measuring the ZBED6 levels. Immunoprecipitation of STAT1 showed that ZBED6 overexpression markedly increased the amount of NEDD4L recruited to STAT1, whereas ZBED6 silencing significantly impaired this recruitment (Figure 6R,S). Functionally, co‐knockdown of STAT1 with ZBED6 significantly reduced AIM2 mRNA and protein levels compared with ZBED6 knockdown alone (Figure S10A–C). STAT1 silencing mitigated ZBED6‐loss‐induced cardiomyocyte death (Figure S10D,E). Taken together, these data suggest that STAT1 acts downstream of ZBED6 in AIM2 regulation.

ZBED6 does not directly bind to the AIM2 promoter but indirectly restrains AIM2 transcription by enhancing STAT1 ubiquitination and proteasomal degradation, limiting STAT1‐driven AIM2 activation and PANoptosis.

### 3.7 ALKBH3 alleviates cardiomyocyte PANoptosis via ZBED6‐dependent suppression of STAT1/AIM2 signaling

**FIGURE 7:**
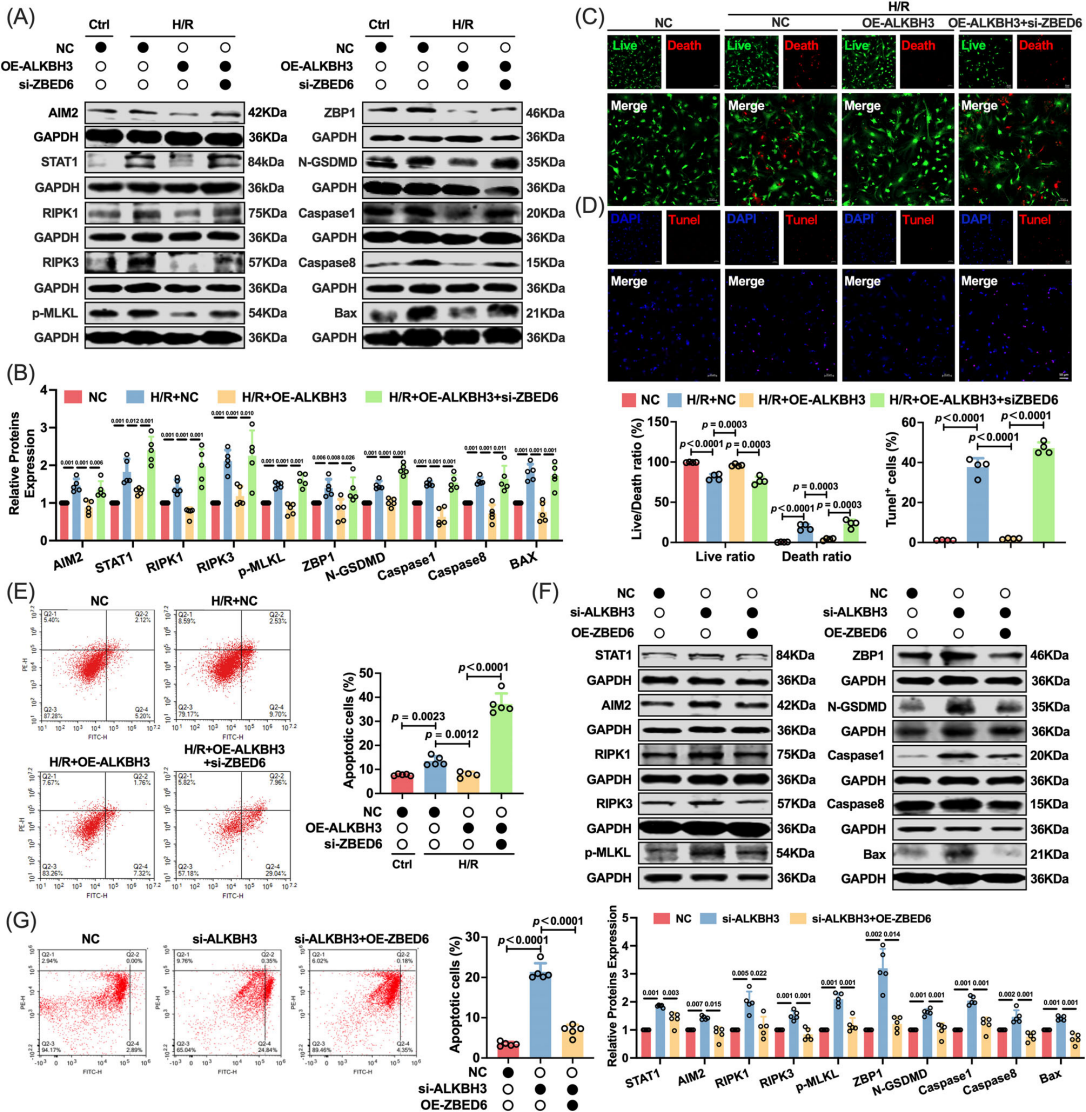
ZBED6 mediates the protective effects of ALKBH3 on cardiomyocyte PANoptosis. (A, B) Western blotting of STAT1 and PANoptosis‐associated proteins (AIM2, RIPK1, RIPK3, p‐MLKL, ZBP1, N‐GSDMD, caspase‐1, caspase‐8 and Bax) in H/R‐treated cardiomyocytes with ALKBH3 overexpression ± ZBED6 knockdown (*n* = 5). (C, D) Live/dead and TUNEL staining (representative images and quantification) under the same conditions (*n* = 4). Magnification: 10×. Scale bar: 50 µm. (E) Annexin V/PI flow‐cytometry analysis of apoptosis in H/R‐treated cardiomyocytes with ALKBH3 overexpression ± ZBED6 knockdown (*n* = 5). (F) Western blotting of PANoptosis‐associated proteins in cardiomyocytes with ALKBH3 knockdown ± ZBED6 overexpression (*n* = 5). (G) Annexin V/PI flow cytometry analysis of apoptosis in H/R‐treated cardiomyocytes with ALKBH3 knockdown ± ZBED6 overexpression (*n* = 5). H/R, hypoxia/reoxygenation; PI, propidium iodide.

To define the hierarchy within the ALKBH3/ZBED6/STAT1/AIM2 axis, reciprocal gain‐ and loss‐of‐function experiments were performed on cardiomyocytes under H/R conditions. ZBED6 silencing partially reversed ALKBH3‐overexpression–mediated suppression of AIM2, STAT1 and PANoptosis‐associated proteins under H/R conditions (Figure 7A,B). Furthermore, the modulatory role of ZBED6 in the ALKBH3‐modulated anti‐PANoptosis response was validated using functional assays. Consistently, ZBED6 knockdown blunted ALKBH3 protection, as shown by the increased injury and death observed in live/dead staining (Figure 7C), TUNEL staining (Figure 7D) and PI flow cytometry (Figure 7E).

Reciprocal experiments were performed using ALKBH3‐silenced cardiomyocytes. As expected, ALKBH3 knockdown markedly upregulated STAT1 expression. ZBED6 overexpression reduced AIM2 and STAT1 levels and attenuated the activation of PANoptosis markers, indicating that ZBED6 is indispensable for the ALKBH3‐conferred resistance to PANoptosis (Figure 7F). This molecular rescue translated into improved cell survival, with fewer PI‐positive cells observed by flow cytometry (Figure 7G). Collectively, these data indicate that ALKBH3 mitigates PANoptosis by upregulating ZBED6, which suppresses STAT1‐dependent AIM2 transcription and downstream PANoptotic signalling. Therefore, ZBED6 is a necessary mediator of ALKBH3‐conferred resistance to PANoptosis during ischemic stress in cardiomyocytes.

**FIGURE 8:**
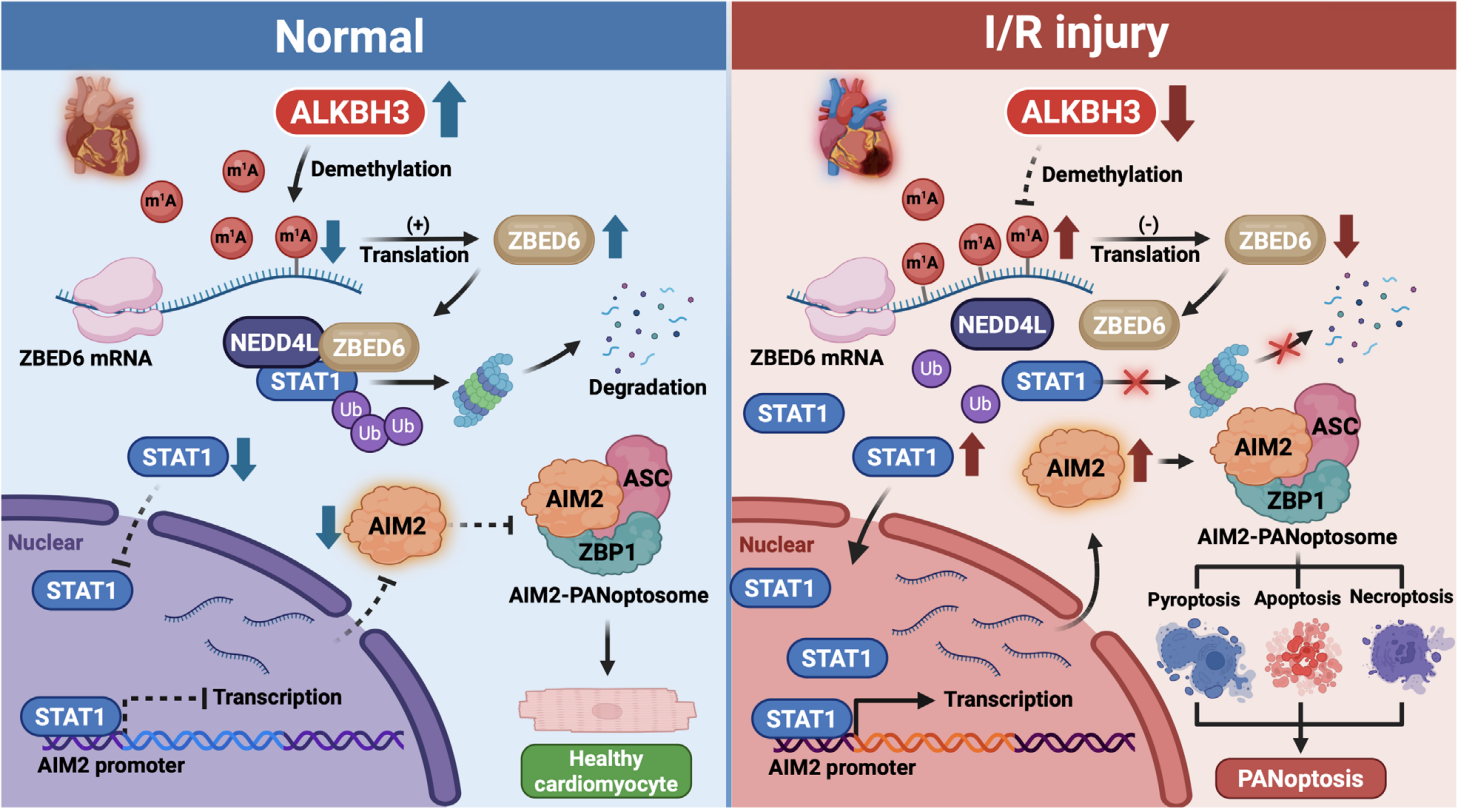
Schematic illustration of the ALKBH3–ZBED6–STAT1–AIM2 axis in myocardial ischemia‐reperfusion injury. Under protective conditions, ALKBH3 functions as an m^1^A demethylase that removes m^1^A modifications from ZBED6 mRNA, enhancing its translation efficiency. Accumulated ZBED6 protein acts as a molecular scaffold to facilitate the recruitment of NEDD4L to STAT1, promoting STAT1 ubiquitination and degradation. This reduction in STAT1 levels suppresses the transcriptional activation of AIM2, consequently inhibiting AIM2‐dependent cardiomyocyte PANoptosis.

A comprehensive mechanism based on these findings is illustrated in Figure 8. As illustrated in the schematic diagram, ALKBH3 removes m^1^A modifications from ZBED6 mRNA to enhance its translation; the accumulated ZBED6 protein then acts as a molecular scaffold to recruit NEDD4L, facilitating STAT1 ubiquitination and degradation, repressing AIM2 transcription and preventing cardiomyocyte PANoptosis, ultimately ameliorating myocardial ischemia‐reperfusion injury.

## 4 DISCUSSION

RNA epigenetic modifications fine‐tune gene expression, and m^1^A has emerged as a crucial regulator of RNA metabolism, including stability, localization and translational efficiency. Unlike the widely studied m^6^A, m^1^A shows dynamic regulation under stressors such as oxidative stress^64^ and inflammation,^65^ both of which are central to myocardial I/R injury. However, the functional relevance of m^1^A in cardiomyocytes remains largely unknown. Here, we identified a cardioprotective mechanism governed by RNA‐modification dynamics and established that ALKBH3, an m^1^A demethylase that selectively removes methyl groups from m^1^A‐modified transcripts,^27^, ^66^ is a key epitranscriptomic regulator of I/R injury. Specifically, ALKBH3 attenuates PANoptosis by suppressing AIM2 transcription via the ZBED6/STAT1 axis, linking RNA methylation to the regulation of innate immune sensors and cell death pathways in the heart. Our data indicate that ALKBH3 affects AIM2 at the transcriptional regulation level via ZBED6 and STAT1, underscoring the importance of m^1^A homeostasis in stress responses. Given the expanding links between m^1^A and disease, pharmacological modulation of RNA‐modifying enzymes, such as ALKBH3, could offer new strategies to curb sterile inflammation and cell death in cardiovascular disorders.

Myocardial I/R injury remains a major clinical challenge, with cardiomyocyte loss driven by ischemia‐induced hypoxia and a secondary wave of inflammation and oxidative stress during reperfusion. Multiple forms of programmed cell death contribute to this process, and growing evidence suggests that PANoptosis, a unified program involving pyroptotic, apoptotic and necroptotic machinery, plays a central role in myocardial damage.^67^, ^68^ However, the upstream regulators of PANoptosis during I/R remain unclear. In this study, we identified ALKBH3 as a critical regulatory checkpoint. Morphologically, transmission electron microscopy provided direct visual evidence that ALKBH3‐deficient cardiomyocytes exhibit a hybrid phenotype characterized by apoptosis, pyroptosis and necroptosis, confirming simultaneous engagement of multiple death pathways. To rigorously validate that ALKBH3 deficiency drives cardiomyocyte death specifically through the PANoptotic program and to rule out potential off‐target effects of the pharmacological inhibitors used in our initial screening, we performed genetic rescue experiments. The observation that specific siRNAs targeting caspase‐1, GSDMD, RIPK3 and MLKL consistently rescued cell viability in ALKBH3‐deficient cardiomyocytes established that ALKBH3 is a key suppressor of PANoptosis. Mechanistically, contrary to a direct demethylation model, our epitranscriptomic mapping did not detect m^1^A peaks in AIM2 mRNA; instead, ALKBH3 indirectly limited AIM2 via ZBED6‐dependent modulation of STAT1. To establish definitive causality, we generated catalytically inactive ALKBH3 mutants (H191A/D193A/H257A) and performed site‐directed mutagenesis of the putative m^1^A sites (A1744 and A1930) within ZBED6 mRNA. Luciferase assays demonstrated that catalytically inactive ALKBH3 mutants failed to enhance ZBED6 translation. Similarly, the A1930G, but not the A1744G, mutation abolished the translational upregulation induced by ALKBH3. These findings indicate that A1930 is a functional m^1^A modification site mapping specifically to the CDS region, and confirm that ALKBH3‐mediated m^1^A demethylation is indispensable for the translational efficiency of ZBED6 mRNA, thereby substantiating the m^1^A‐based mechanism of translational control. Although AIM2 has been linked to inflammasome activation during I/R injury and atherosclerosis,^69^, ^70^ its epigenetic regulation and contribution to cardiomyocyte PANoptosis during I/R have not been sufficiently defined. Consistent with recent reports of PANoptosis in I/R,^44^, ^45^, ^71^ we showed that AIM2 was markedly upregulated during I/R and that cardiomyocyte‐specific AIM2 deletion improved cardiac injury. AIM2 activation coincides with the engagement of pyroptotic (caspase‐1 and GSDMD), apoptotic (caspase‐8 and BAX) and necroptotic (RIPK1, RIPK3 and p‐MLKL) effectors, positioning AIM2 as the central regulator of PANoptosis.

An additional mechanistic insight is the identification of the ZBED6/STAT1 signalling axis. ZBED6 is a classically conserved DNA‐binding transcriptional repressor that recognizes promoter motifs to restrain genes involved in growth, development and metabolism^72^, ^73^, ^74^ Our study uncovered a noncanonical paradigm for ZBED6 function that operates independently of its DNA‐binding activity. We demonstrated that ZBED6 can be repurposed as a molecular scaffold that orchestrates the post‐translational modification of STAT1. We showed that ZBED6 directly interacts with STAT1 and functions as a molecular bridge to recruit the E3 ubiquitin ligase NEDD4L. Mechanistically, MD simulations and interaction assays revealed that ZBED6 is essential for stabilizing the ternary complex between STAT1 and NEDD4L, enhancing their interaction efficiencies. This protein–protein interaction‐based mechanism promotes STAT1 ubiquitination, accelerates its turnover and limits STAT1‐dependent AIM2 transcription by reducing STAT1 occupancy at target loci. STAT1 is a core mediator of interferon signalling and immune responses^75^ and has been implicated in inflammatory damage,^5^ apoptosis,^76^ necroptosis^77^ and adverse remodelling via the induction of downstream proinflammatory genes.^78^ Although interferon signalling has been linked to PANoptosis,^79^, ^80^ a direct link between STAT1 and AIM2 transcription in cardiomyocytes has not been established. Our data demonstrate that STAT1 binds to the AIM2 promoter and activates its transcription during I/R, linking interferon/STAT1 signalling to inflammasome priming and PANoptosis induction. Collectively, these results expand the transcriptional regulation landscape of AIM2 in the heart and indicate that ZBED6 is a context‐dependent modulator of TF stability and not merely a static DNA‐binding repressor. In summary, ALKBH3 enhances ZBED6 expression via m^1^A demethylation, ZBED6 promotes STAT1 ubiquitination and degradation mediated by NEDD4L, and reduced STAT1 limits AIM2 transcription, constituting the ALKBH3/ZBED6/STAT1/AIM2 cascade that inhibits inflammatory cell death.

This study had several limitations that warrant further discussion. Regarding the upstream regulation of ALKBH3, the exact mechanisms governing its downregulation during I/R require further investigation. Given the sensitivity of DNA repair enzymes to cellular metabolic states, it is plausible that the acute burst of oxidative stress and hypoxic microenvironment during I/R directly compromises ALKBH3 stability. In addition, we focused on m^1^A‐dependent effects using m^1^A‐specific MeRIP‐seq under ALKBH3 overexpression, which clarified one mechanism but did not exclude broader roles. ALKBH3 has also been implicated in the removal of other epigenetic markers, including DNA alkylation,^63^ and may crosstalk with other RNA modifications. Functionally, besides the specific regulation of ZBED6, ALKBH3 plays a pivotal role in maintaining genomic integrity. Its downregulation likely creates a cellular environment characterized by DNA replication stress. Although our study confirmed that AIM2 is a key sensor, ALKBH3 deficiency may also engage parallel DNA‐sensing mechanisms, such as the cGAS‐STING pathway. Therefore, the ALKBH3‐ZBED6‐STAT1‐AIM2 axis is a critical component of the epitranscriptomic stress response. Another critical consideration is the cellular specificity of AIM2 signalling. Consistent with previous reports, AIM2 is upregulated in both cardiomyocytes and infiltrating immune cells following ischemia. Although our study used cardiomyocyte‐specific genetic tools (Myh6‐Cre mice and cTNT‐promoter AAV) to rigorously confirm the intrinsic protective role of the ALKBH3/ZBED6/STAT1/AIM2 axis within cardiomyocytes, we cannot exclude the possibility that ALKBH3‐mediated regulation of AIM2 in infiltrating immune cells may also contribute to an inflammatory microenvironment. Unravelling the potential intercellular crosstalk between cardiomyocytes and immune cells mediated by AIM2 signalling represents an intriguing direction for future studies. Finally, although we demonstrated that ZBED6 promotes STAT1 ubiquitination and degradation, it remains unclear whether ZBED6 also exerts transcriptional control of STAT1. ChIP and promoter‐reporter assays were used to determine whether ZBED6 exerts additional transcriptional control over STAT1. Elucidating these multilayered regulatory mechanisms will provide a more comprehensive understanding of cardiac stress responses. Targeting the ALKBH3‐ZBED6‐STAT1 axis could pave the way for precise therapies designed to reduce excessive inflammation in patients with ischemic heart disease.

In conclusion, this study broadens the functional repertoire of ALKBH3 and reveals a mechanistic link between epitranscriptomic regulation and inflammatory PANoptosis induction. By integrating m^1^A‐dependent RNA control with TF signalling and inflammasome activation, we identified the ALKBH3/ZBED6/STAT1/AIM2 axis, which coordinates multiple cell death pathways in ischemic hearts. These insights provide a conceptual and mechanistic foundation for therapeutic strategies that target this cascade to simultaneously restrain pyroptosis, apoptosis and necroptosis during myocardial I/R.

## AUTHOR CONTRIBUTIONS

*Data curation, investigation, visualization and writing—original draft*: Hongtao Diao, Chunlei Wang and Yuting Xiong. *Investigation and visualization*: Qiaoyue Zhao, Xinyue Zhang, Xiaohui Qi, Yuan Zou and Jiaxuan Li. *Formal analysis and supervision*: Linghua Zeng, Wei Si and Feng Zhang. *Supervision*: Ping Pang and Ning Wang. *Conceptualization and writing—review and editing*: Yu Bian. Conceptualization, resources and funding acquisition: Baofeng Yang.

## CONFLICT OF INTEREST STATEMENT

The authors declare no conflict of interest.

## ETHICS STATEMENT

This study complied with the regulations of the Experimental Animal Ethics Committee of Harbin Medical University (Permit number: IRB3026722). The protocol was approved by the experimental animal ethics committee of Harbin Medical University.

## Supporting information



Supporting Information

Supporting Information

## Data Availability

The datasets generated and/or analyzed during the current study are available from the corresponding author upon reasonable request.
