# Supplementary material for: ALKBH3 suppresses ischemia/reperfusion‐induced PANoptosis by regulating the ZBED6/STAT1/AIM2 axis through m1A demethylation
Source: Clin Transl Med. 2026 Mar 12;16(3):e70632. doi: 10.1002/ctm2.70632 (PMC13093621; doi:10.1002/ctm2.70632)
Supplement: Supplementary file 1 — Supporting Information [file CTM2-16-e70632-s001.docx]

**ALKBH3 suppresses ischemia/reperfusion****–induced PANoptosis by inhibiting the ZBED6/STAT1/AIM2 axis through m¹A demethylation**

Hongtao Diao^1,*^, Chunlei Wang^2,*^, Yuting Xiong^2,*^, Qiaoyue Zhao^2^, Xinyue Zhang^2^, Xiaohui Qi^2^, Yuan Zou^2^, Jiaxuan Li^2^, Linghua Zeng^2^, Wei Si^2^, Feng Zhang^2^, Ping Pang^3^, Ning Wang^2,#^, Yu Bian^2,#^, Baofeng Yang^1,2,#^.

**^1^**Department of Pharmacology, College of Basic Medical Sciences, Jilin University, Changchun 130021, China.

**^2^**Department of Pharmacology, the State Key Laboratory of Frigid Zone Cardiovascular Diseases (SKLFZCD), Key Laboratory of Cardiovascular Research, Ministry of Education, College of Pharmacy, Harbin Medical University, Harbin 150081, China.

**^3^**Shanghai Frontiers Science Research Center for Druggability of Cardiovascular Noncoding RNA, Institute for Frontier Medical Technology, College of Chemistry and Chemical Engineering, Shanghai University of Engineering Science, Shanghai 201620, China.

^#^Corresponding author: Baofeng Yang, Yu Bian, Ning Wang.

E-mail addresses:

Baofeng Yang: [yangbf@ems.hrbmu.edu.cn](mailto:yangbf@ems.hrbmu.edu.cn); Yu Bian: [bianyu@hrbmu.edu.cn](mailto:bianyu@hrbmu.edu.cn) and Ning Wang: [wangning@ems.hrbmu.edu.cn](mailto:wangning@ems.hrbmu.edu.cn).

^*^These authors contributed equally: Hongtao Diao, Chunlei Wang, and Yuting Xiong.

Running title: ALKBH3 suppresses cardiomyocyte PANoptosis during I/R injury.

**Supplementary tables**

**Table S1. Abbreviations**

| **Abbreviation** | **Full name** |
| --- | --- |
| ALKBH3 | AlkB homolog 3, RNA demethylase |
| PANoptosis | Pyroptosis, apoptosis, and necroptosis |
| ZBED6 | Zinc finger BED-type containing 6 |
| STAT1 | Signal transducer and activator of transcription 1 |
| AIM2 | Absent in Melanoma 2 |
| I/R | Ischemia/reperfusion |
| m^1^A | mRNA N¹-methyladenosine |
| siRNA | Small interfering RNA |
| RIP | RNA immunoprecipitation |
| ChIP-qPCR | Chromatin immunoprecipitation–quantitative PCR |
| PCI | Percutaneous coronary intervention |
| LAD | Left anterior descending coronary artery |
| AAV | Adeno-associated virus |
| NMVCs | Neonatal mouse ventricular cardiomyocytes |
| H/R | Hypoxia/reoxygenation |
| CHX | Cycloheximide |
| ActD | Actinomycin D |
| EF | Ejection fraction |
| FS | Fractional shortening |
| LVID | Left ventricular internal diameter |
| TTC | 2,3,5-Triphenyltetrazolium chloride |
| TUNEL | Terminal deoxynucleotidyl transferase dUTP nick end labeling |
| PI | Propidium iodide |
| H&E | Hematoxylin and eosin |
| Bax | Bcl-2–associated X protein |
| RIPK1 | Receptor-interacting serine/threonine-protein kinase 1 |
| RIPK3 | Receptor-interacting serine/threonine-protein kinase 3 |
| MLKL | Mixed lineage kinase domain-like protein |
| Caspase-1 | Cysteine-aspartic protease 1 |
| Caspase-8 | Cysteine-aspartic protease 8 |
| GAPDH | Glyceraldehyde-3-phosphate dehydrogenase |
| Co-IP | Co-immunoprecipitation |
| ChIP | Chromatin immunoprecipitation |
| CCK-8 | Cell Counting Kit-8 |
| SEM | Standard error of the mean |
| GEO | Gene Expression Omnibus |

**Table S2. siRNA sequences used in this study**

| **Target gene** | **Sense (5′→3′)** | **Antisense (5′→3′)** |
| --- | --- | --- |
| ALKBH3 | GGACCUUGCUAAUCAUGGATT | UCCAUGAUUAGCAAGGUCCTT |
| AIM2 | GCAGUGACAAUGACUUUAA | UUAAAGUCAUUGUCACUGC |
| ZBED6 | CCGCCAGAUACUACAAGAATT | UUCUUGUAGUAUCUGGCGGTT |
| MLKL | GGCAAUGAUAAGAUCCUCUTT | AGAGGAUCUUAUCAUUGCCTT |
| CASPASE1 | GGACCACAUACUCUAAUGATT | UCAUUAGAGUAUGUGGUCCTT |
| RIPK3 | GCUCUGGUGACAAGAUUCATT | UGAAUCUUGUCACCAGAGCTT |
| GSDMD | GCUGCAGACAAAGGAGGAATT | UUCCUCCUUUGUCUGCAGCTT |
| STAT1 | CUGCCUAUGAUGUCUCGUUTT | AACGAGACAUCAUAGGCAGTT |

**Table S3. Construct maps of plasmids**

| **Target gene/protein** | **Construct map** |
| --- | --- |
| ALKBH3 | 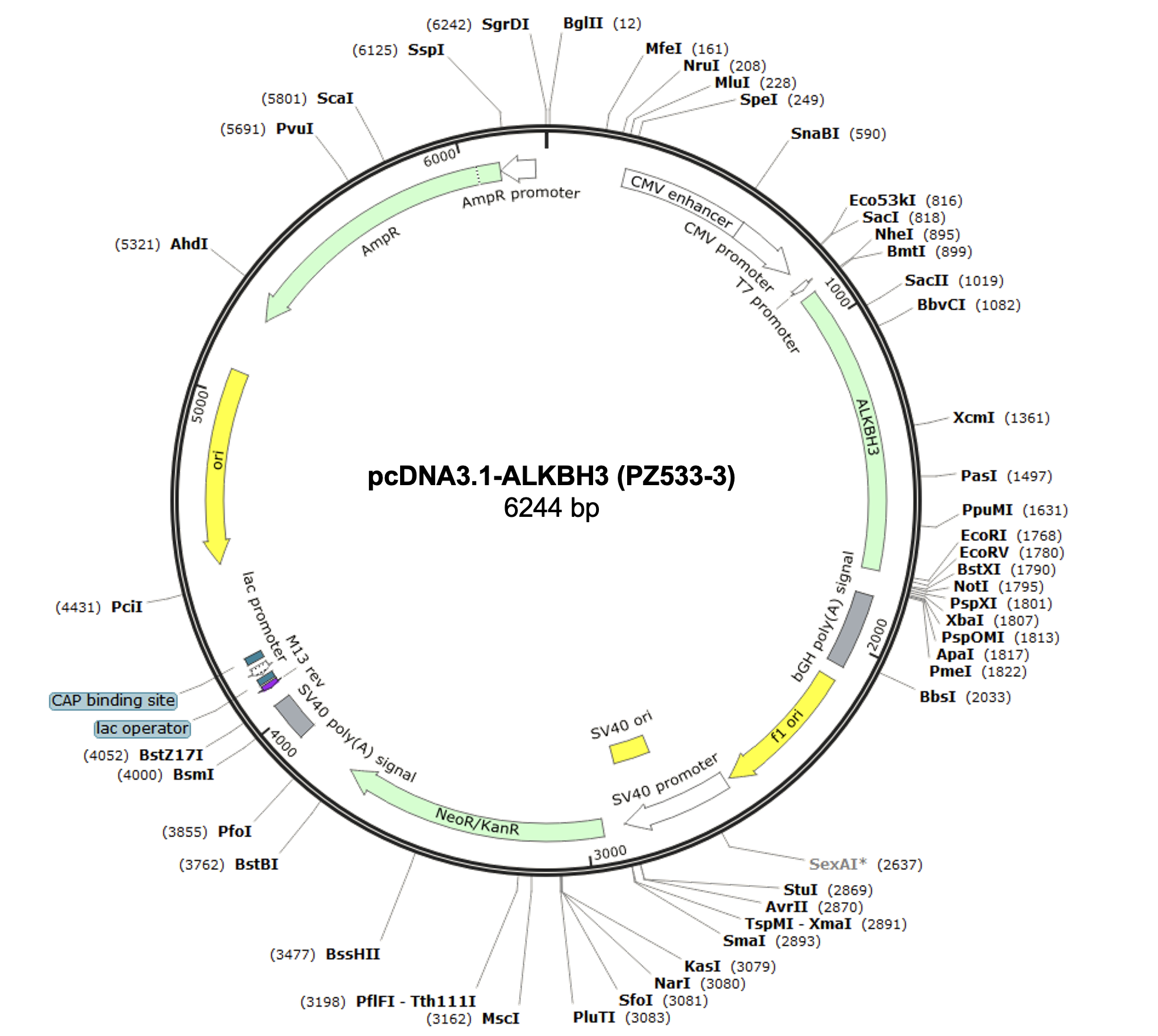 |
| ZBED6 | 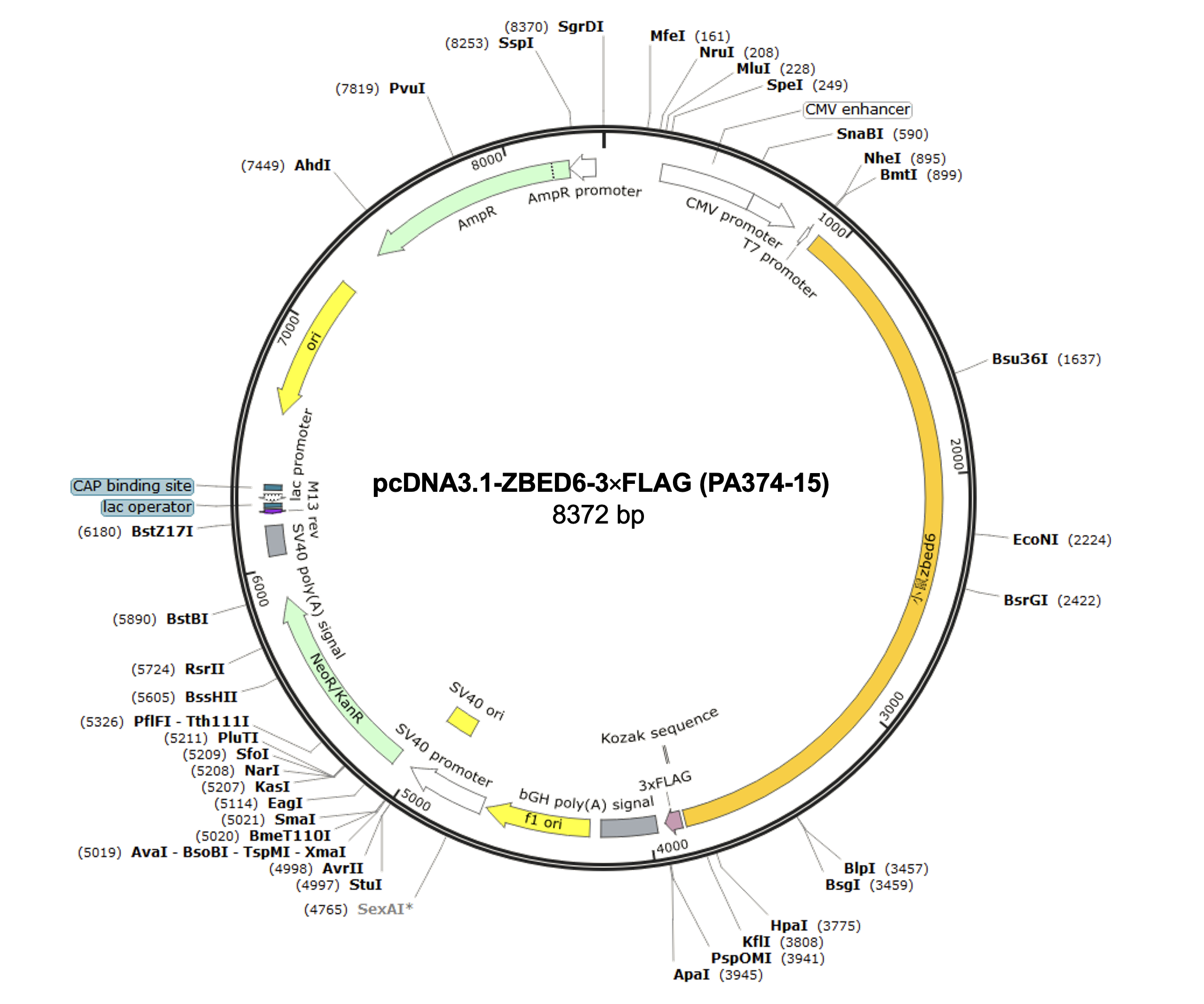 |

**Table S4. Inhibitors used in this study**

| **Name** | **Manufacturer** | **Catalog number** |
| --- | --- | --- |
| z-VAD | KKL Med Inc. | KM9980 |
| Nec-1 | KKL Med Inc. | KM3145 |
| Disulfiram | KKL Med Inc. | KM12553 |
| CQ | MCE, MedChemExpress | HY-17589A |
| MG132 | MCE, MedChemExpress | HY-13259 |
| ActD | MCE, MedChemExpress | HY-17559 |
| CHX | MCE, MedChemExpress | HY-12320 |

**Table S5. Antibodies used in this study**

| **Antibody** | **Manufacturer** | **Catalog number** | **Dilution ratio** |
| --- | --- | --- | --- |
| GAPDH | ZSBG-BIO | 390035 | 1:2000 |
| ALKBH3 | Proteintech | 12292-1-AP | 1:1000 |
| AIM2 | ABclonal | A3356 | 1:1000 |
| ZBED6 | Atlas Antibodies | HPA068807 | 1:1000 |
| STAT1 | Proteintech | 10144-2-AP | 1:1000 |
| BAX | Proteintech | 50599-2-Ig | 1:1000 |
| Caspase-1 | Proteintech | 22915-1-AP | 1:1000 |
| RIPK1 | Proteintech | 29932-1-AP | 1:1000 |
| RIPK3 | Proteintech | 17563-1-AP | 1:1000 |
| phospho-MLKL | Proteintech | 82090-2-RR | 1:1000 |
| Caspase-8 | Proteintech | 13423-1-AP | 1:1000 |
| N-GSDMD | Bioworld | BS79581 | 1:500 |
| ZBP1 | ABclonal | A13899 | 1:1000 |
| NEDD4L | Proteintech | 13690-1-AP | 1:1000 |

**Table S6. Primer sequences for ChIP-qPCR and Aim2 promoter luciferase assays**

| **Assay** | **Primer name** | **Sequence (5′→3′)** |
| --- | --- | --- |
| ChIP–qPCR | AIM2-Promoter-F | AAAGGGTAGCTTTGAAAGAGGGT |
| ChIP–qPCR | AIM2-Promoter-R | ATTGCTTTTGTCCCACTCCTCA |
| Luciferase (STAT1 5′-deletion) | AIM2-1MluI-F | CGACGCGTTAAGATCTAGACGTCAGATGGGAAGGAGAA |
| Luciferase (STAT1 5′-deletion) | AIM2-2MluI-F | CGACGCGTAAATGACGTCTCTGATTGGCCATTTAAGGA |
| Luciferase (STAT1 5′-deletion) | AIM2-3MluI-F | CGACGCGTTGCTGTGTGTGGAGACCAAGGATGTGGAAA |
| Luciferase (STAT1 5′-deletion) | AIM2-4MluI-F | CGACGCGTCACGGGATGCTGGAGGGACAGGGAGAAGCC |
| Luciferase (STAT1 5′-deletion) | AIM2-XhoI-R | CCGCTCGAGGGTATCGTGTCTGCTCTTAACCAGCTCAGA |
| Luciferase (ZBED6 motif mutant) | AIM2-mut-1F | GCTAGACTCCTGAATCTCTGTCGACTCTCACTGAGTCACTGAGTCTGTGC |
| Luciferase (ZBED6 motif mutant) | AIM2-mut-1R | AGTGAGAGTCGACAGAGATTCAGGAGTCTAGCTTGAAAACTTAAGAGAAT |
| Luciferase (ZBED6 motif mutant) | AIM2-mut-2F | GAGCTTCGTGGGTAAATCAATGATAGTGGGCGGCAGTGTAAACACCCTCC |
| Luciferase (ZBED6 motif mutant) | AIM2-mut-2R | GCCGCCCACTATCATTGATTTACCCACGAAGCTCTTTTAGAGCTTTATCT |

**Supplementary figures**

**Figures S1**


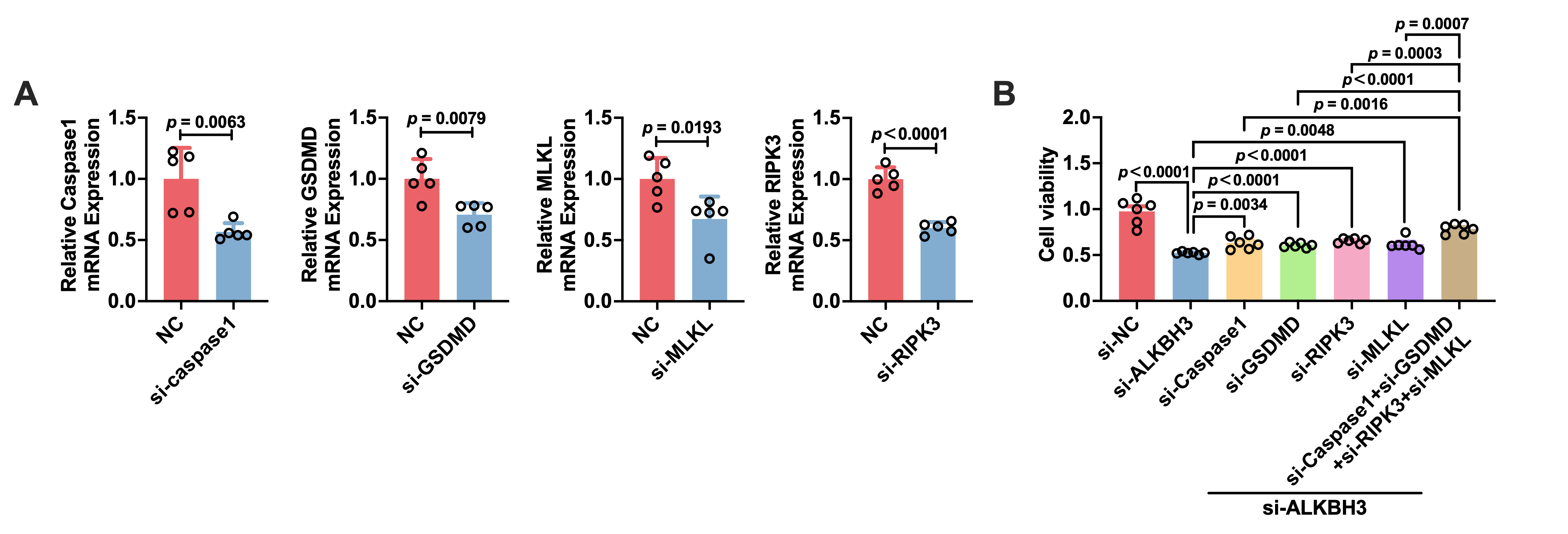


**Figure S1**. **Knockdown of Caspase1, GSDMD, RIPK3, MLKL partially ameliorates the injury induced by si-ALKBH3.** (A) ﻿qRT-PCR analyzed the knockdown efficiency of siRNAs targeting caspase1, GSDMD, MLKL, RIPK3 in cardiomyocytes (n = 5) (B) Cell viability following ALKBH3 knockdown and transfection with siRNAs targeting downstream effectors (Caspase1, GSDMD, RIPK3, MLKL), individually or in combination (n = 6).

**Figure S2**


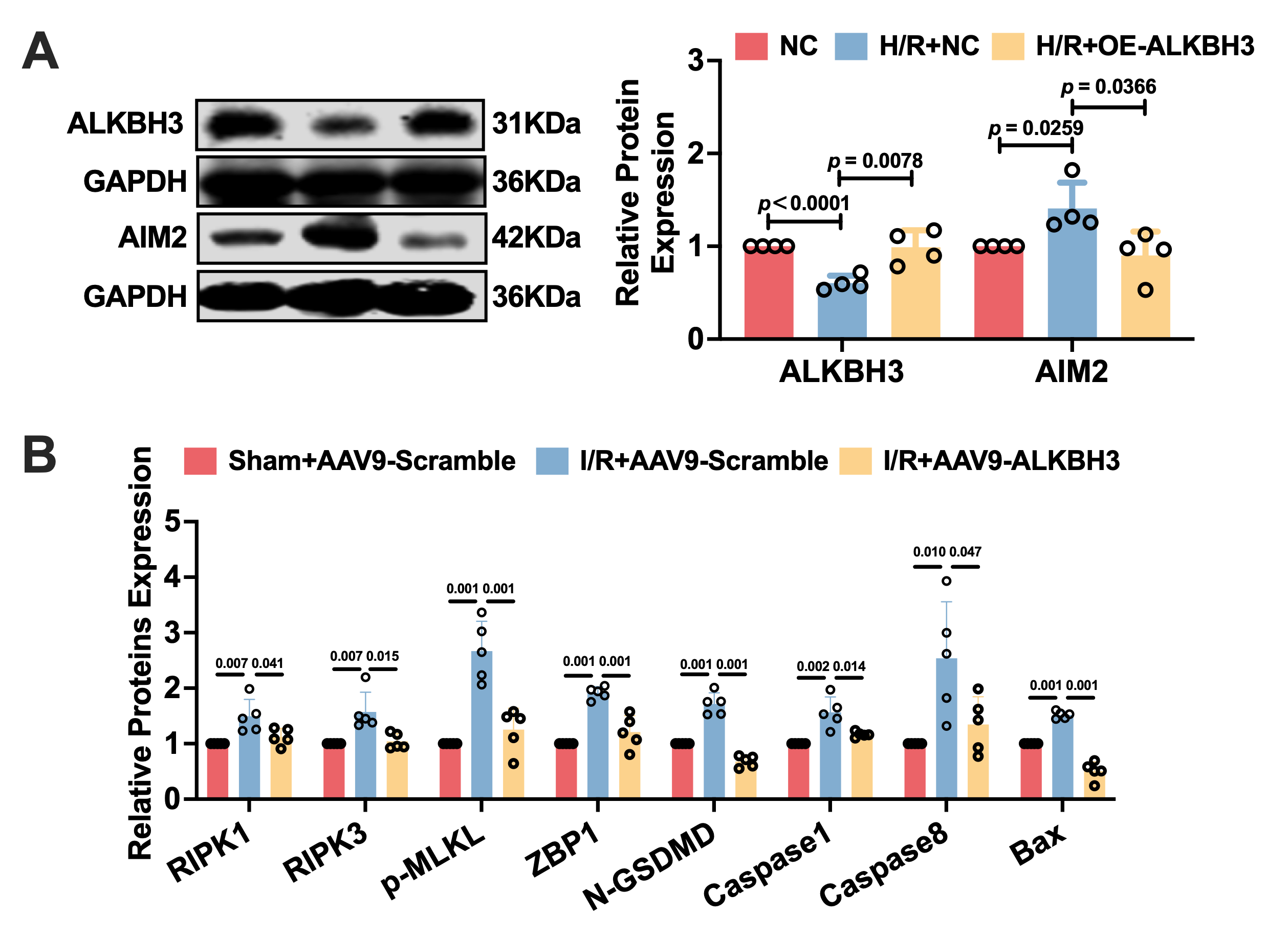


**Figure S2**. **Overexpression of ALKBH3 suppresses AIM2 expression and PANoptosis-related protein levels.** (A) Western blot analysis of ALKBH3 and AIM2 expression in AC16 cells (n = 4). (B) Quantification of RIPK1, RIPK3, p-MLKL, ZBP1, N-GSDMD, Caspase1, Caspase8 and Bax expression in mouse myocardial tissue (n = 5).

**Figure S3**


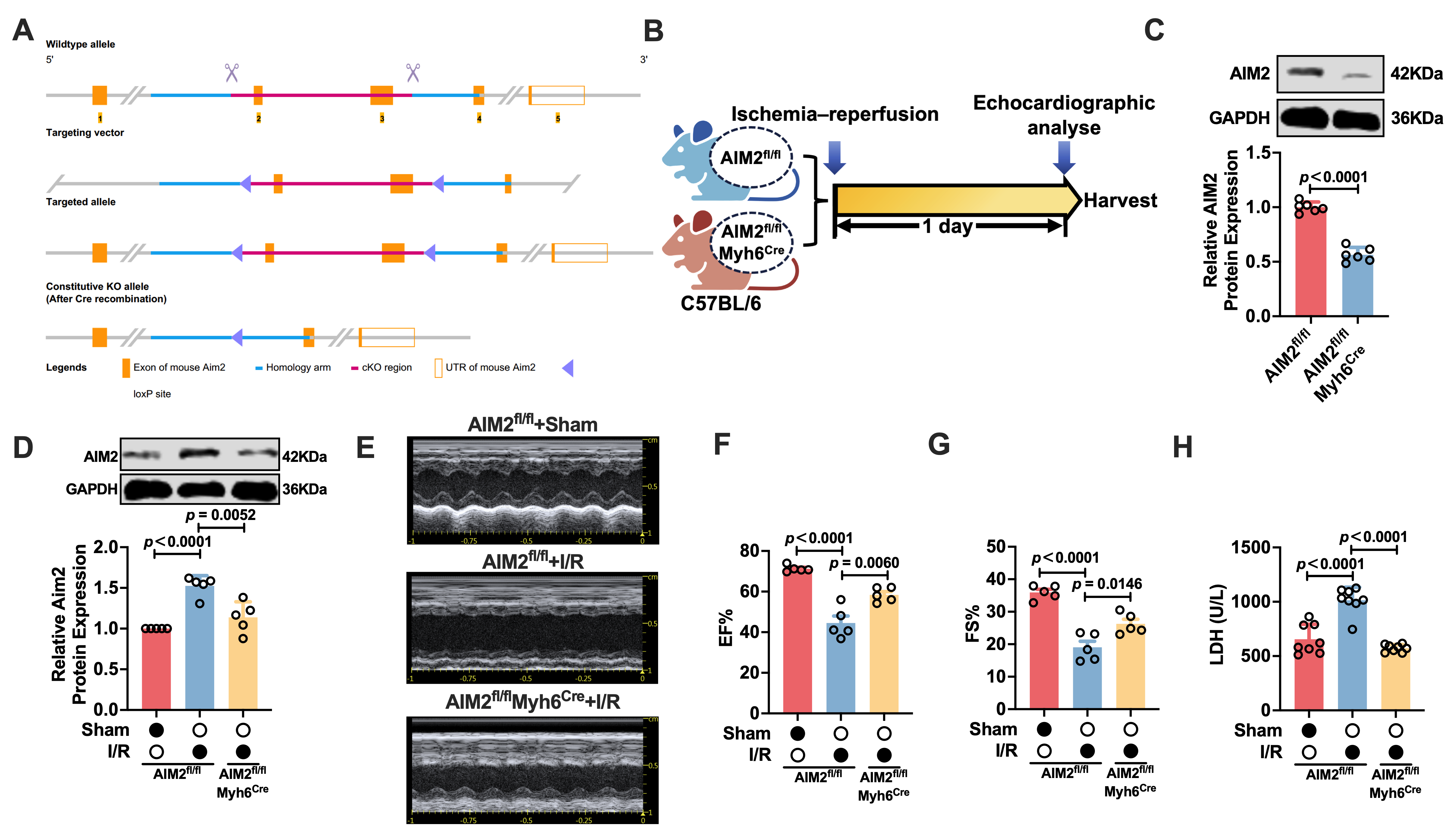


**Figure S3**. **Cardiomyocyte-specific knockout of AIM2 improves myocardial I/R injury.** (A) ﻿Schematic diagram for the construction of cardiomyocyte AIM2 knockout mice. AIM2^fl/fl^ mice were crossed with Myh6^Cre^ to obtain AIM2^fl/fl^ Myh6^Cre^ mice. (B) Schematic diagram showing that the I/R model was established in wild type or cardiomyocyte-specific AIM2 knockout mice. (C, D) Western blot analysis of AIM2 expression in mouse myocardial tissue (n = 5-6). (E–G) Representative images of echocardiographs and assessment of left ventricular EF% and FS% (n = 5). (H) Serum levels of LDH (n = 8).

**Figure S4**


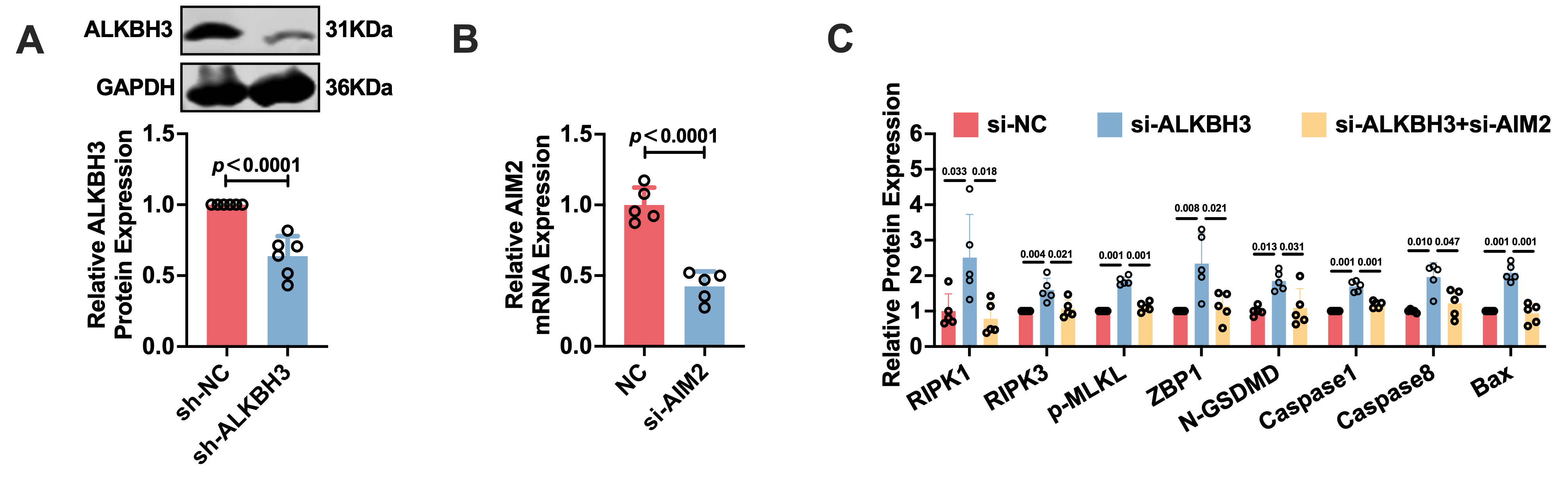


**Figure S4. AIM2 knockdown attenuates upregulation of PANoptosis-related protein levels induced by ALKBH3 silencing.** (A) Western blot analyzed the knockdown efficiency of AAV9-shALKBH3 in mouse myocardial tissue (n = 6). (B) qRT-PCR analyzed the knockdown efficiency of siRNA-AIM2 in cardiomyocytes (n = 5). (C) Quantification of RIPK1, RIPK3, p-MLKL, ZBP1, N-GSDMD, Caspase1, Caspase8, and Bax in cardiomyocytes (n = 5).

**Figure S5**


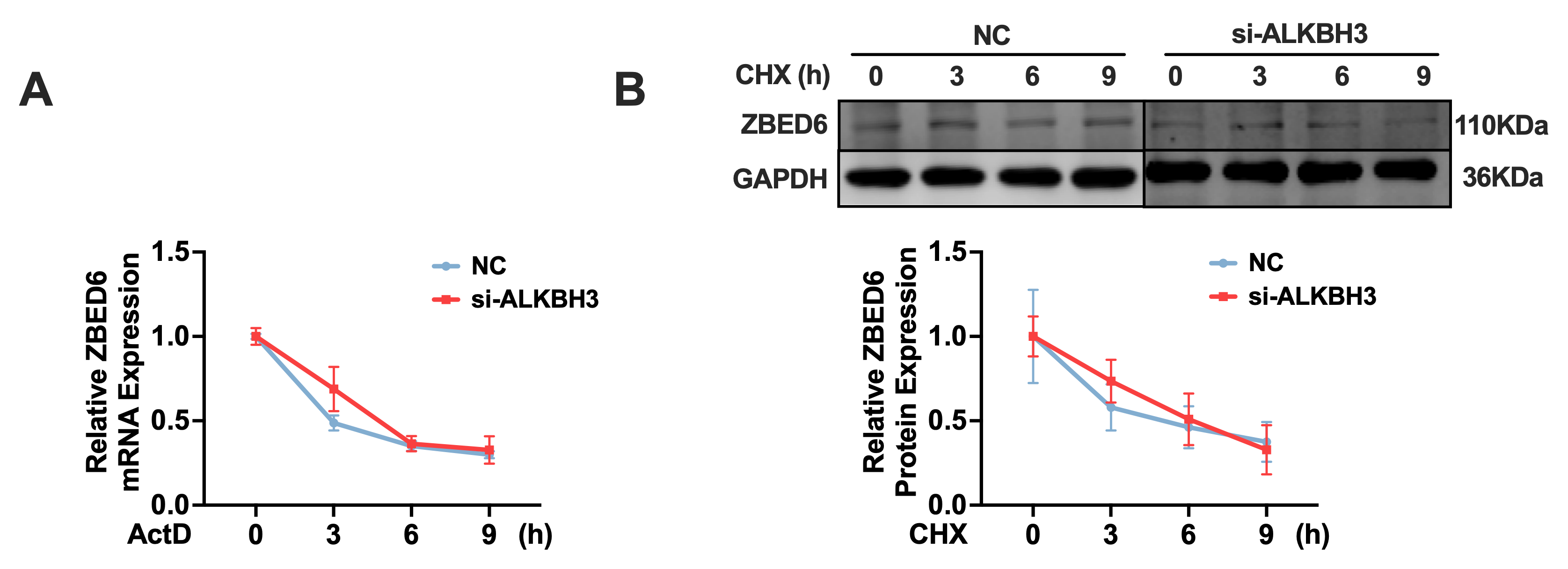


**Figure S5. Knockdown of ALKBH3 does not alter the stability of ZBED6 mRNA and protein.** (A) Actinomycin D (ActD) chase assay. Cardiomyocytes transfected with control siRNA or si-ALKBH3 were treated with ActD and harvested at 0, 3, 6, and 9 h for qRT–PCR analysis of ZBED6 mRNA (n = 5). (B) Cycloheximide (CHX) chase assay. Cardiomyocytes transfected with control siRNA or si-ALKBH3 were treated with CHX and harvested at 0, 3, 6, and 9 h for western blot analysis of ZBED6 protein (n = 5).

**Figure S6**


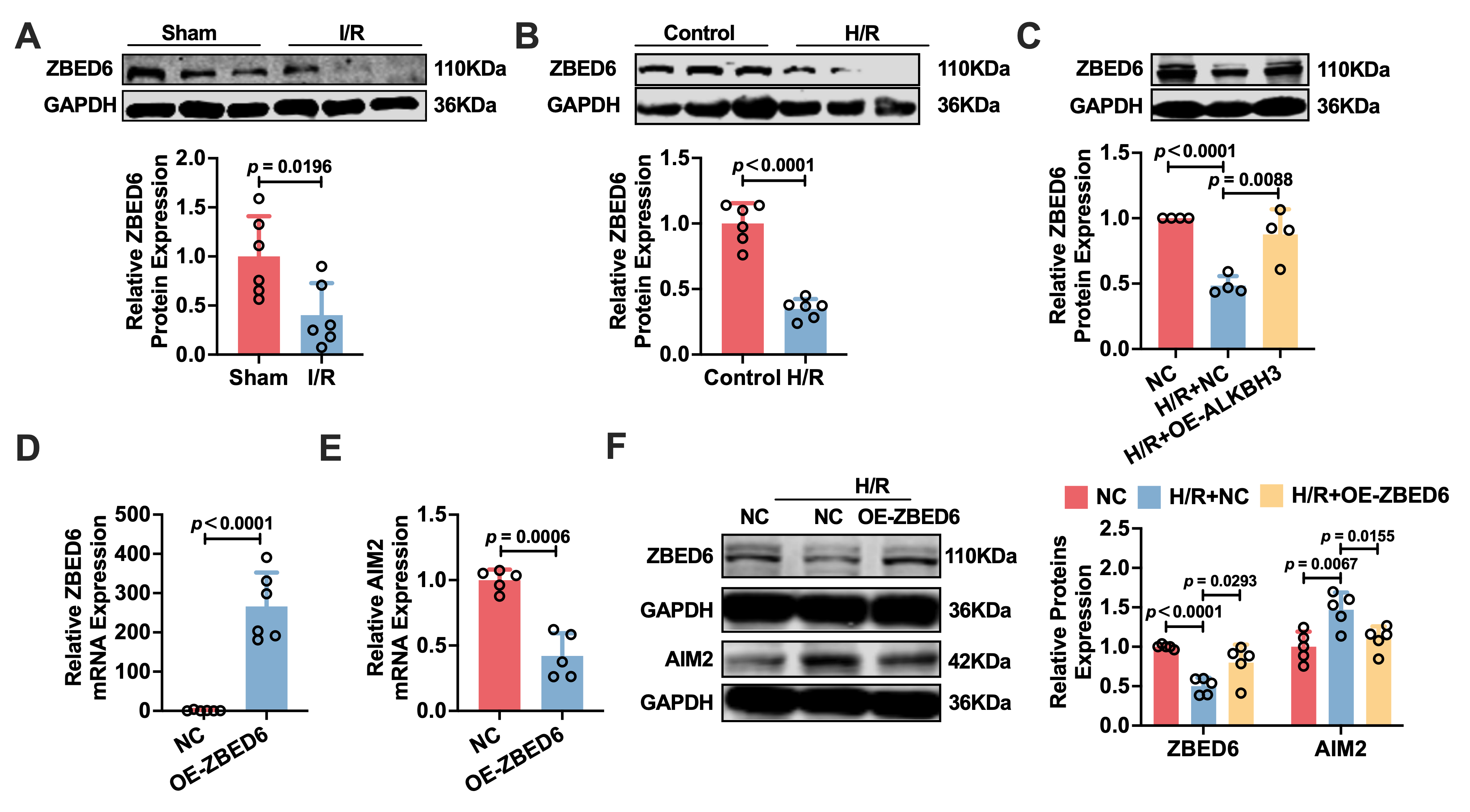


**Figure S6. ZBED6 suppresses AIM2 expression in cardiomyocytes.** (A) Western blot analysis of ZBED6 expression in mouse myocardial tissue from the Sham and I/R groups (n = 6). (B) Western blot analysis of ZBED6 expression in cardiomyocytes under normoxic or H/R conditions (n = 5). (C) Western blot analysis of ZBED6 expression in AC16 cells under H/R conditions with or without ALKBH3 overexpression (n = 4). (D) qRT-PCR analysis confirming ALKBH3 overexpression in cardiomyocytes (n = 6). (E) qRT-PCR analyzed AIM2 mRNA expression upon ZBED6 overexpression (n = 5). (F) Western blot analysis of ZBED6 and AIM2 expression in cardiomyocytes under H/R conditions with or without ZBED6 overexpression (n = 5).

**Figure S7**


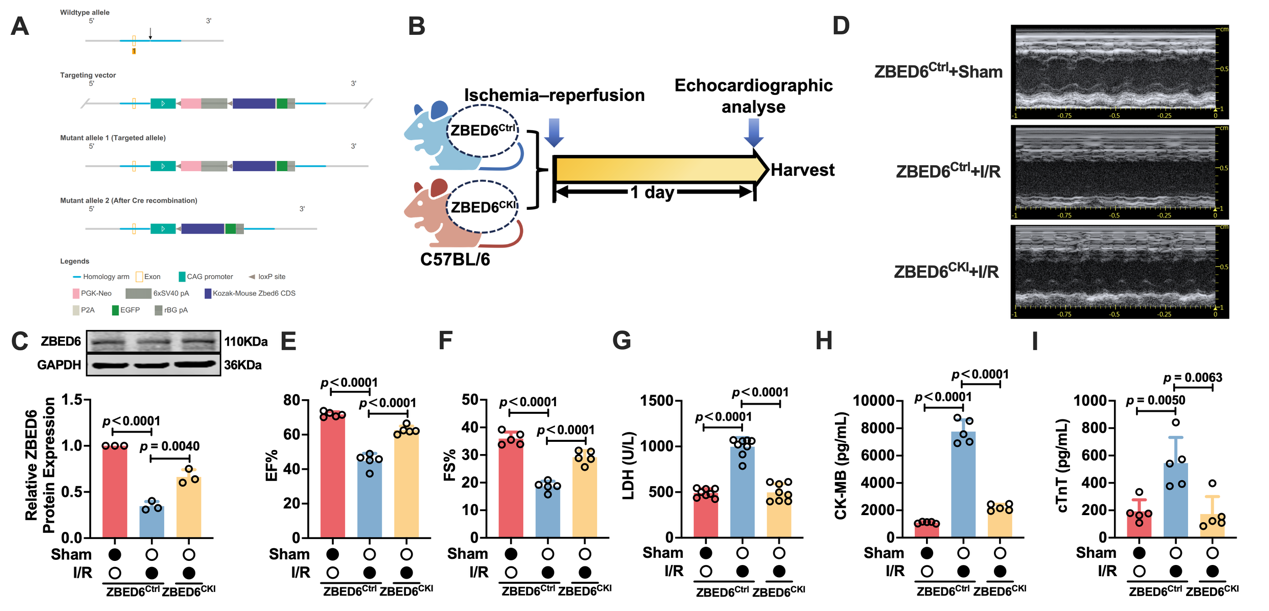


**Figure S7**. **Cardiomyocyte-specific knock-in of ZBED6 improves myocardial I/R injury.** (A) ﻿Schematic diagram for the construction of cardiomyocyte ZBED6 knock-in mice. Rosa26-LSL-ZBED6 mice were crossed with Myh6^Cre^ to obtain ZBED6^CKI^ mice. (B) Schematic diagram showing that the I/R model was established in wild type or cardiomyocyte-specific ZBED6 knock-in mice. (C) Western blot analysis of ZBED6 expression in mouse myocardial tissue (n = 3). (D–F) Representative images of echocardiographs and assessment of left ventricular EF% and FS% (n = 5). (G-I) Serum levels of LDH, CK-MB, and cTnT (n = 5).

**Figure S8**


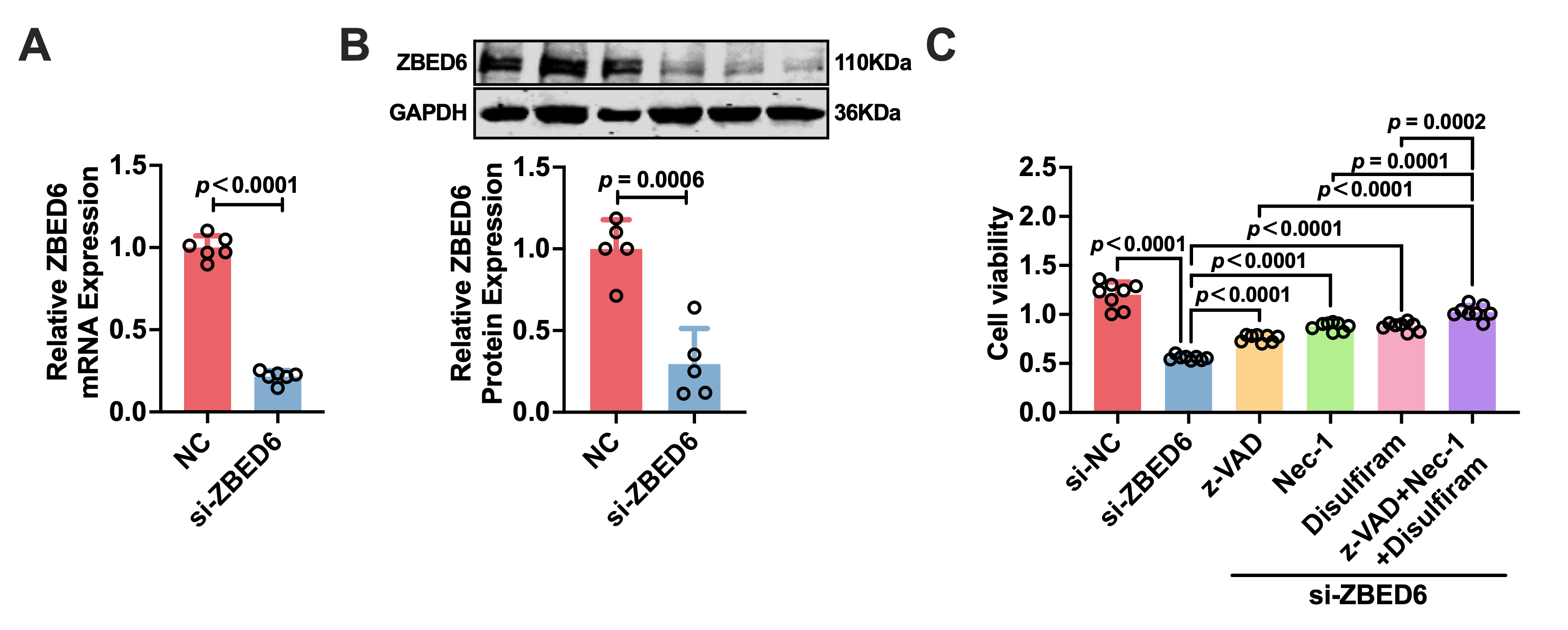


**Figure S8. Knockdown of ZBED6 induces cardiomyocyte PANoptosis.** (A, B) qRT-PCR and western blot analyzed the knockdown efficiency of siRNA-ZBED6 in cardiomyocytes (n = 5-6). (C) Cell viability following ALKBH3 knockdown and treatment with individual or combined pathway inhibitors (z-VAD, Nec-1, disulfiram) (n = 8).

**Figure S9**


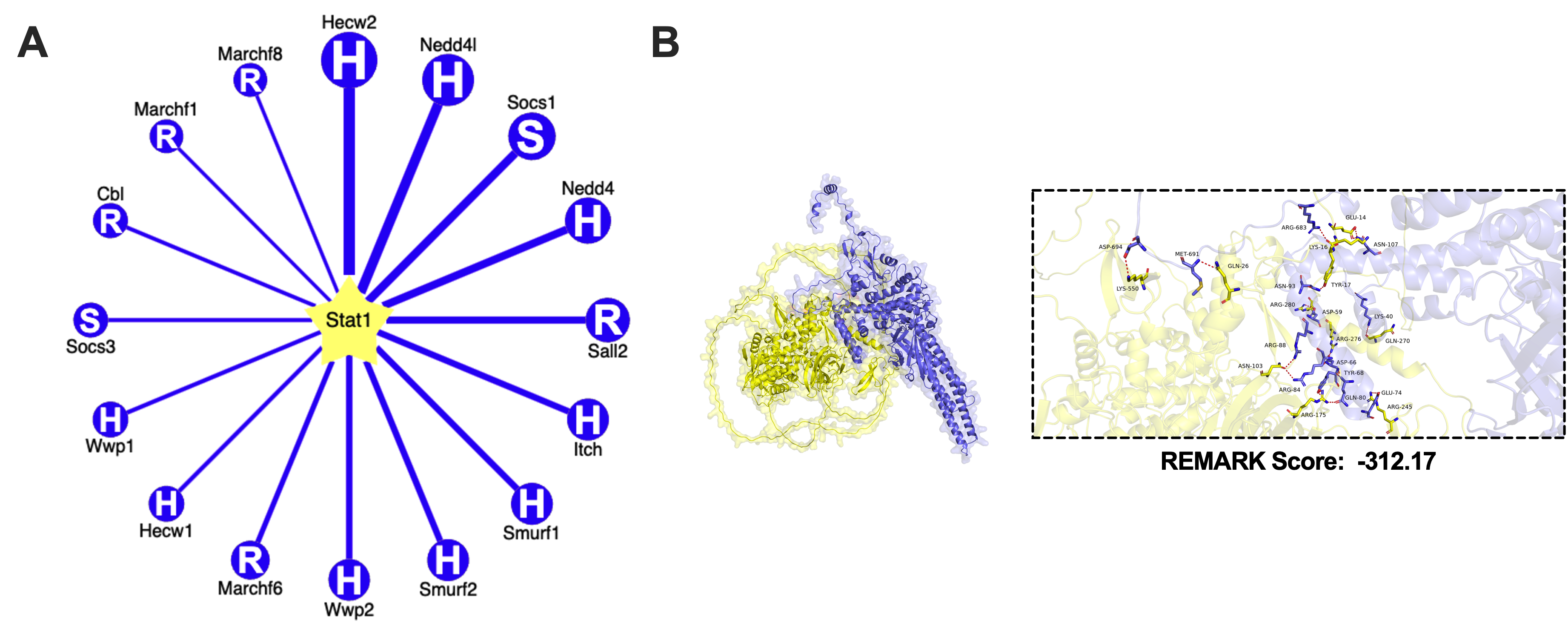


**Figure S9. An in silico screen is performed to identify E3 ubiquitin ligases that interact with STAT1.** (A) Website prediction of ubiquitin enzymes associated with STAT1. (B) Molecular docking model of the STAT1–NEDD4L interaction.

**Figure S10**


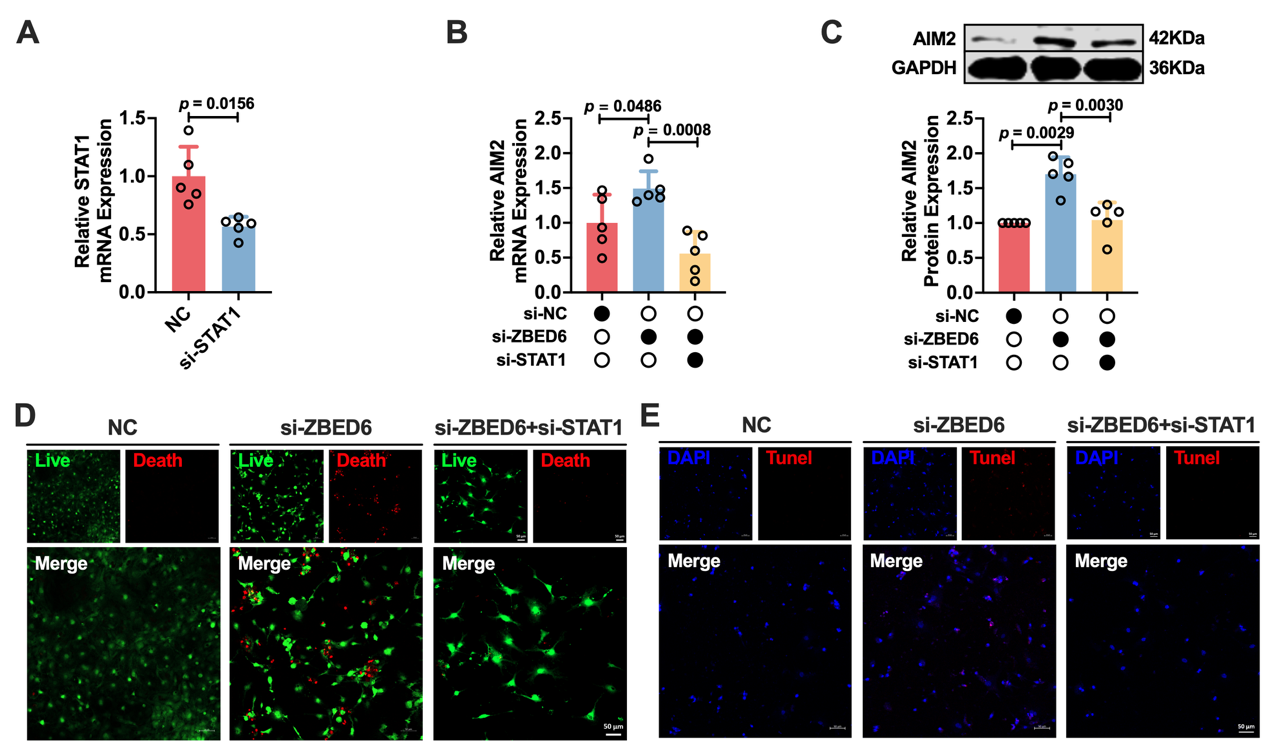


**Figure S10. Knockdown of STAT1 suppresses cardiomyocytes death induced by ZBED6 silencing.** (A) qRT-PCR analyzed the knockdown efficiency of siRNA-STAT1 in cardiomyocytes (n = 5). (B, C) qRT-PCR and western blot analysis of AIM2 expression in cardiomyocytes (n = 5). (D) Live/dead cell staining and (E) TUNEL staining upon knockdown of ZBED6 and STAT1 (n = 4). Magnification: 10×. Scale bar: 50 µm.
